# Unseen and underserved: A scoping review of perinatal mental health interventions in Sub-Saharan Africa

**DOI:** 10.1371/journal.pmen.0000739

**Published:** 2026-10-01

**Authors:** Basil H. Aboul-Enein, Suha Ballout, Patricia J. Kelly

**Affiliations:** 1 College of Arts & Sciences, Health & Society Program, University of Massachusetts Dartmouth, North Dartmouth, Massachusetts, United States of America; 2 Faculty of Public Health and Policy, London School of Hygiene & Tropical Medicine, London, United Kingdom; 3 School of Nursing, University of Washington, Seattle, Washington, United States of America; 4 College of Nursing, Thomas Jefferson University, Philadelphia, Pennsylvania, United States of America; PLOS: Public Library of Science, UNITED KINGDOM OF GREAT BRITAIN AND NORTHERN IRELAND

## Abstract

Maternal mental health (MMH) remains a critical public health issue across Sub-Saharan Africa (SSA). Despite the high burden, access to culturally appropriate and evidence-based mental health interventions remains limited. This scoping review examines interventions targeting mental health outcomes for pregnant and postpartum women across SSA countries. Guided by PRISMA-ScR and Arksey and O’Malley’s framework, a comprehensive search was conducted across 14 electronic databases for peer-reviewed intervention studies published in English between 2000 and March 2026. A total of 57 studies across 18 SSA countries were identified with interventions primarily focused on the individual or mother-infant level. The majority used strong research methods, with widely varying intervention content, including cognitive-behavioral therapy, problem-solving therapy, psychoeducation, and community-based programs, often delivered by community health workers, focusing on problem-solving skills, and involving cash transfers. While most studies reported reductions in depressive symptoms and improved psychosocial outcomes, few addressed scalability, health system integration, or cost-effectiveness. Few studies worked with health professionals or included technology interventions. A wide range of mental health interventions show promise in addressing perinatal mental health in SSA. However, gaps remain in scaling up sustainable, culturally relevant, and system-integrated interventions. Future efforts should prioritize provider education, health system strengthening, and evaluation of economic impact to enhance MMH outcomes across the region. To have a significant impact on perinatal mental health, future interventions that focus on cost-effectiveness, scalability, health professions education, and integration of technology are needed.

## Introduction

Maternal mental health is an urgent yet under-addressed public health priority in Sub-Saharan Africa (SSA). This region is made up of over 45 countries located south of the Sahara Desert [1,2]. The countries are marked by shared structural challenges such as high fertility rates, weak health systems, widespread poverty, and distinct sociocultural norms that influence reproductive and mental health outcomes. Cultural values include strong pronatalist values, patriarchal family structures, and cultural expectations that discourage expressions of emotional vulnerability [3].

Mental health conditions during the perinatal period, particularly depression and anxiety, are prevalent and impair maternal functioning, infant development, and overall family wellbeing [4]. Recent data indicate that perinatal depression affects between 10% and 40% of women in SSA, with some subpopulations, including adolescent mothers, reporting rates as high as 60% [3–6]. The mental health burden is disproportionately high among those experiencing intersecting vulnerabilities such as poverty, HIV, unplanned pregnancies, intimate partner violence (IPV), and social exclusion [7–9].

Despite the burden, maternal mental health remains neglected in most national policy frameworks and underfunded within health system budgets [7]. Cultural and social determinants exacerbate the invisibility of maternal mental disorders. In many SSA contexts, motherhood is idealized, and mental distress is perceived as a sign of weakness, spiritual imbalance, or moral failing [10–12]. This stigma prevents disclosure and delays help-seeking [7,13] Traditional beliefs and patriarchal norms restrict women’s autonomy over their health decisions, further impeding access to care [11,14,15].

A variety of intervention models have emerged to address maternal mental health, ranging from peer-led psychosocial support groups and community health worker training to integrated antenatal care screening and mobile health (mHealth) tools [16]. Task-shifting strategies, including training of lay health workers and integration with HIV or Prevention of Mother-To-Child Transmission (PMTCT) services, have shown promise in resource-limited settings [17,18]. However, interventions vary significantly by setting, and evidence on their effectiveness, scalability, and cultural relevance remains fragmented [17].

This scoping review fills a literature gap by examining the breadth of maternal mental health interventions across SSA, with attention to intervention types, populations served, implementation contexts, and reported outcomes. By synthesizing this evidence and situating it within broader social, cultural, and policy contexts, the review aims to inform future research, program design, and policy reform to better support mothers’ mental wellbeing across the region.

### Background

To understand the current intervention landscape, it is essential to contextualize the epidemiology and sociocultural framing of maternal mental health in SSA. Maternal mental health challenges in SSA are shaped by the intersecting determinants of biological, social, economic, and structural factors [6,7]. Among the most common conditions are perinatal depression, anxiety, post-traumatic stress disorder (PTSD), and suicidal ideation, all of which adversely impact maternal wellbeing and child health and development [11,14]. Epidemiological data show alarming prevalence rates, with studies across Ghana, Uganda, Cameroon, Ethiopia, and South Africa documenting postpartum depression rates ranging from 20% to over 50% [19,20]. In Cameroon, for example, nearly half of pregnant women reported depressive symptoms during antenatal care visits [5,9,20], and in Uganda, rates of depression were particularly high among adolescent mothers and those with limited social support [7,21].

These mental health burdens are embedded in broader structural and sociocultural contexts. Poverty, food insecurity, early marriage, unplanned pregnancy, and gender-based violence have consistently been identified as major risk factors for perinatal mental illness [8,9]. Women with HIV infection face heightened psychological distress due to their health status and social stigma, especially when services are siloed or lack continuity of care [22–24]. In Benin, women who experienced severe obstetric complications reported higher rates of psychological distress, particularly when compounded by financial hardship or intimate partner abuse [9,22].

Cultural interpretations of mental health also profoundly shape maternal experiences and pathways to care in SSA. In many communities, maternal distress is not framed as a biomedical issue but interpreted through spiritual or moral lenses, leading women to seek traditional or religious remedies [7,13]. These beliefs often coexist with patriarchal expectations that women should endure suffering in silence to fulfill idealized maternal roles, thereby deterring open discussion or help-seeking [15,25].

Despite the magnitude of the issue, most SSA countries lack formal mental health policies or that explicitly include maternal mental health [16]. Where policies exist, they are often poorly implemented due to underfunding, lack of trained personnel, and centralized urban services inaccessible to rural populations [4]. Overwhelmed health systems frequently prioritize infectious disease control and maternal mortality reduction over mental health integration, leaving a critical gap in comprehensive maternal care [3,8]. The result is a systemic underinvestment in maternal mental health screening, referral, and treatment services, particularly in primary care settings [16]. These intersecting social, cultural, and structural challenges underscore the urgency of scalable and contextually grounded interventions.

Addressing maternal mental health in SSA therefore requires not only expanding interventions but also transforming how maternal distress is conceptualized within health systems and communities. A multi-layered approach integrating biomedical, psychosocial, and culturally responsive perspectives offers the most promising path toward building inclusive, resilient maternal health systems and advancing intergenerational wellbeing. To capture the breadth and depth of existing approaches and identify critical gaps in knowledge and practice, this scoping review maps maternal mental health interventions implemented across SSA.

## Methods

A scoping review methodology, rather than a systematic review with meta-analysis, was selected because our objective was to map the breadth of maternal mental health interventions implemented across SSA, spanning heterogeneous intervention types (individual psychotherapy, community health worker-delivered programs, economic interventions, technology-based tools), populations, study designs, and outcome measures, rather than to estimate a pooled effect size for a single, narrowly defined intervention-outcome comparison [26,29]. This approach follows established guidance that scoping reviews are indicated when a research question is broad and exploratory, or when the evidence base is expected to be heterogeneous in design and reporting, precluding meaningful quantitative synthesis [26].

### Literature search

Consistent with the methodological approaches used in previously published regional scoping reviews on maternal mental health interventions [26,27], this scoping review of the literature was conducted using 14 databases, applying the PRISMA Extension for Scoping Reviews and the Arksey and O’Malley framework [28,29]. A combination of search terms and phrases related to mental health, pregnant and postpartum women, living in the 48 countries located south of the Sahara Desert (**see** Table 1). For example, the full search strategy applied in PubMed combined the following terms: (“depression” OR “mental health” OR “mental wellbeing” OR “psychosocial” OR “psychosocial resilience” OR “distress”) AND (“pregnant” OR “postpartum” OR “antenatal” OR “prenatal” OR “gravid”) AND (“Intervention” OR “program” OR “programme” OR “promotion” OR “promoting”) AND (the name of each of the 48 countries located south of the Sahara Desert, e.g., “Angola” OR “Benin” OR  ... OR “Zimbabwe”; full list in Table 1), limited to English-language, peer-reviewed journal articles published between 2000 and March 2026. Database-specific search adaptations, reflecting each platform’s indexing conventions, are detailed in Table 2, and the full search and screening flow is summarized in Fig 1.

**Table 1:** PICOS Criteria for inclusion and exclusion of studies.

Parameter	Inclusion Criteria	Exclusion Criteria
**Population**	• Pregnant and postpartum female populations residing in Sub-Saharan Africa*	• Sub-Saharan African diaspora
**Intervention type**	Any form of non-pharmacological intervention that address mental health-related outcomes, including: • Community-based programs • Family-based programs • MHealth/digital tools • Educational interventions • Environmental Interventions	• Pharmacological interventions alone • Interventions that do not address mental health-related outcomes.
**Comparators**	• Control: received no intervention. • Received partial intervention (e.g., educational intervention only vs. multi-componential intervention.)	N/A
**Outcomes of interest**	• changes in mental health outcomes • changes in mental health-related knowledge • changes in mental health referrals	• Non-mental health related outcomes
**Language**	English	All other languages
**Study Type**	• Intervention studies with measured outcomes. • Peer-reviewed original research articles • Original research conference publications	• Non-numeric/categorical assessments or qualitative studies • Non-peer-reviewed articles • Study protocols • Commentaires • Narratives Communications • Non-intervention studies • White papers • Similar article types • Grey literature • Thesis/Dissertations

* Angola; Benin; Botswana; Burkina Faso; Burundi; Cabo Verde; Cameroon; Central African Republic; Chad; Comoros; Congo; Cote d’Ivoire; Democratic Republic of the Congo; Djibouti; Equatorial Guinea; Eritrea; Eswatini; Ethiopia; Gabon; The Gambia; Ghana; Guinea; Guinea- Bissau; Kenya; Lesotho; Liberia; Madagascar; Malawi; Mali; Mauritania; Mauritius; Mozambique; Namibia; Niger; Nigeria; Rwanda; Sao Tome and Principe; Senegal; Seychelles; Sierra Leone; Somalia; South Africa; South Sudan; Togo; Tanzania; Uganda; Zambia; Zimbabwe

**Abbreviations:** N/A – Not applicable

**Table 2:** Electronic Databases Used with Relevant Search Period and Terms.

Databases	Search Period	Keywords, search terms, and phrases
ArticleFirst; Biomed Central; BioOne; BIOSIS; CINAHL; EBSCOHost; ProQuest; PubMed; SAGE Reference Online; ScienceDirect; Scopus; SpringerLink; Taylor & Francis; and Wiley Online	2000 and February 2026	**(All fields)** “depression” OR “mental health” OR “mental wellbeing” OR “psychosocial” OR “psychosocial resilience” OR “distress” AND **(All fields)** “pregnant” OR “postpartum” OR “antenatal” OR “prenatal” OR “gravid” AND **(All fields)** “Intervention” OR “program” or “programme” OR “promotion” OR “promoting” AND **(All fields)** “Angola”; “Benin”; “Botswana”; “Burkina Faso”; “Burundi”; “Cabo Verde”; “Cameroon”; “Central African Republic”; “Chad”; “Comoros”; “Congo”; “Cote d’Ivoire”; “Democratic Republic of the Congo”; “Djibouti”; “Equatorial Guinea”; “Eritrea”; “Eswatini”; “Ethiopia”; “Gabon”; “Gambia”; “Ghana”; “Guinea”; “Guinea- Bissau”; “Kenya”; “Lesotho”; “Liberia”; “Madagascar”; “Malawi”; “Mali”; “Mauritania”; “Mauritius”; “Mozambique”; “Namibia”; “Niger”; “Nigeria”; “Rwanda”; “Sao Tome and Principe”; “Senegal”; “Seychelles”; “Sierra Leone”; “Somalia”; “South Africa”; “South Sudan”; “Togo”; “Tanzania”; “Uganda”; “Zambia”; “Zimbabwe”

**Fig 1:**
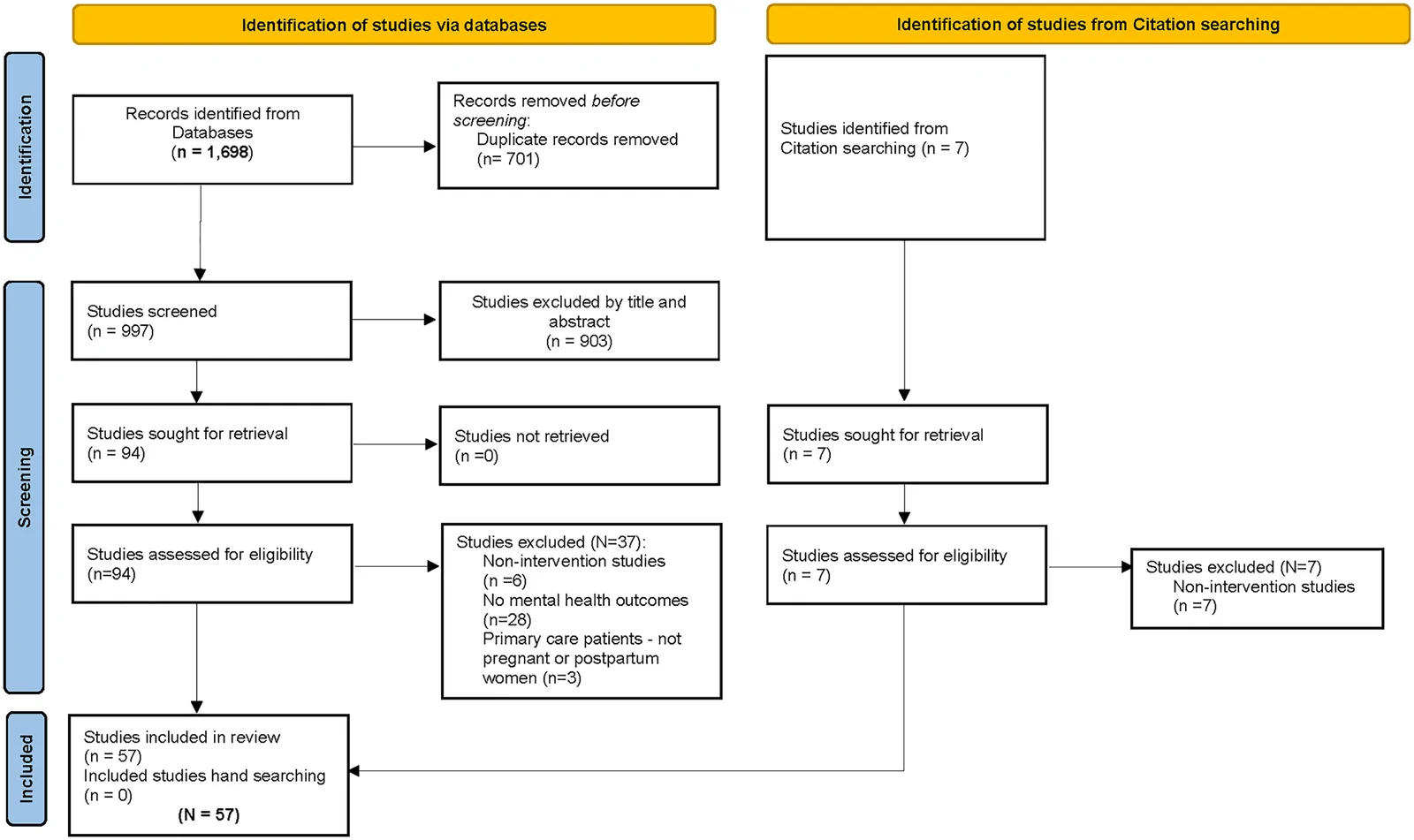
2020 PRISMA ScR flow diagram.

Reference lists of retrieved articles were hand-searched for additional eligible studies, and all records identified were screened against the eligibility criteria below.

### Eligibility criteria

Eligible studies were intervention-based, published in English in peer-reviewed journals between 2000 and March 2026, and reported mental health-related outcomes as either a primary or secondary/component outcome of a multi-level intervention (individual, community, family, or policy), among pregnant women and/or mothers in SSA who received an educational, training, or other intervention. We excluded protocol papers, studies conducted outside SSA, non-English-language publications, brief communications, and grey literature or non-peer-reviewed sources (**see** Table 2).

### Study selection and data extraction

One author conducted the full literature search and performed initial screening and selection of studies using Rayyan QCRI [30]. Where inclusion was uncertain, a second author was consulted, and disagreements between the two were resolved through discussion. For data extraction, the 57 included studies were divided among three authors, each of whom extracted the following for their assigned subset: author, date, target population, country, type of study, sample size, intervention details, measured parameters, main results, and main recommendations (see Table 3). One author then cross-checked all extracted data across the full set of studies for consistency and accuracy; discrepancies were resolved through team discussion. As methodological quality assessment is not a prerequisite for scoping reviews, we did not appraise the methodology of the included studies [29].

**Table 3:** Summary Findings (N = 57).

Author, Year, Country	Aims & Objectives	Research Design	Sample Size, Population	Intervention	Key Findings		Recommendations
Agler et al. (2021); Democratic Republic of Congo	Quantify association of implementing BFH Steps on PPD; test if association mediated by breastfeeding difficulties	Cluster RCT	903 mother-baby pairs from 6 hospitals (2 implementing Steps 1-9; 2 implementing Steps 1-10; 2 providing SOC	*Baby-Friendly Hospital Initiative:* Steps 1-9 vs. Steps 1-10 (includes community groups to provide ongoing BF source of support & knowledge beyond what hospital staff provides at follow-up visits	Depressive symptoms reduced in the groups implementing BFHI Steps 1–9 or 1–10 relative to SOC, with Steps 1–10 associated with largest decrease		Implementation of BFH steps associated with reduction in depression; implementation globally may be helpful in mitigating negative impact of PPD
Bah et al. (2025); Sierra Leone	Assess feasibility, acceptability, preliminary effectiveness of intervention delivered by mother-to-mother support groups	RCT with 2 group waitlist	39 PN women; intervention: 30; comparison: 19	*Problem-Solving Therapy:* *Friendship Bench Intervention*: mother-to-mother support groups provided 4 weeks of home-based, individual problem-solving therapy, followed by a peer-led group session (circle of serenity)	Intervention proved feasible & acceptable; post-depression scores significantly lower in intervention than control group (p < 0.05)		Given the promising preliminary results, RCTs recommended to confirm effectiveness, guide nationwide implementation
Balogun et al. (2023); Angola	Assess effect of handbook intervention package on MCH health outcomes for mother and child	Cluster RCT	9039 pregnant women; intervention: 3774; comparison: 5265	*MCH Handbook*: 3 parts: 1) distribution of handbook that covers maternal, newborn, child health; 2) provider training, 3) community education among pregnant women on use of handbook	No difference between groups in maternal behavior-based continuum of care at three months PP; no statistically significant difference in MH outcomes between groups		Distribution of MCH handbook & supplementary interventions did not increase service use sufficiently to meet study goals
Bam et al. (2026); Ghana & Uganda	Assess feasibility & effectiveness of interprofessional education program on PMH	Mixed methods	60 students with 71.7% response rate	Interactive teaching, 1day, 5-hour program with faculty from midwifery, medicine, nursing on mental well-being; epidemiology, types, PMH risk/protective factors; screening tools, MH assessments; interventions, coping mechanisms;	Participants perceived program as well organized (95.3%), easy to understand (90.7%), positive experience; improvements in all areas, including knowledge, attitudes, behavior, teamwork, communication, potential application in practice, enhanced self-efficacy, skills		Demonstrates effectiveness of interprofessional education on PMH, with improved knowledge, attitudes, teamwork, communication; recommend more engagement time, earlier curriculum integration
Baumgartner et al. (2021) Ghana	Assess impact of the lay counselor- delivered, group program on MH of mothers of children under age 2 & on child development	Cluster RCT	374 pregnant women; intervention: 221; comparison:153	*Integrated Mothers & Babies Course & Early Childhood* *Development:* CBT content 17 sessions over 7 months, delivered by “model mothers” to help mothers manage realities via application of select coping skills; key messages of antenatal care, breastfeeding, seeking timely care for sick child	No significant effects on MH or child development		Real-world evaluation had null findings; post-intervention depression levels were very low in both arms (3%); future work should examine potential impact of women’s groups on postpartum MH with more varied content
Bengtson et al. (2023); Malawi	Evaluate feasibility, acceptability, fidelity, preliminary efficacy of intervention to improve PPD, HIV care engagement	2-group pilot RCT	80 pregnant women; intervention: 40; comparison: 40	*Enhanced Friendship Bench:* 4 antenatal & 2 PP counseling sessions using 6-step process of problem-solving therapy	Finding of strong feasibility, acceptability & fidelity; at 6 months PP, intervention group had significantly improved depression remission, retention in HIV care, viral suppression compared to usual care		Intervention should be evaluated in fully powered RCT to evaluate efficacy for PPD, engagement in HIV care
Bitew et al. (2025); Ethiopia	Evaluate feasibility, fidelity, & acceptability of problem-solving therapy for AN depression	RCT	50 pregnant women with depression symptoms; intervention: 25; comparison: 25	South African model of problem-solving therapy; 4 sessions, women identified important things and problems in their lives, categorized them, planned how to address	Intervention feasible & acceptable; more training of intervention team needed;		Training needed for intervention team to practice skills
Bliznashka et al. (2021); Tanzania	Evaluate effect of CHW delivered home visit, health intervention & cash transfers on maternal depressive symptoms	Cluster RCT	593 pregnant women/ mothers with child <12 months; 3 arms: CHW: 200; CHW & cash transfer: 200; control:193	CHW-delivered info on essential early child development knowledge, age-appropriate play, communication, toy making, parenting & problem-solving, caregiver responsiveness	CHW intervention significantly reduced depression scores compared with control; cash transfers lowered scores but results not statistically significant; some positive effects on maternal responsiveness to infant		Intervention reduced maternal depressive symptoms; mechanisms of action unknown; cash transfers appeared to provide limited to no additional benefit
Bliznashka et al. (2026); Malawi	Evaluated the impact of nutrition-sensitive social behaviour change intervention (SBC) with or without cash transfers on maternal PN depression during the lean season in rural Malawi	Secondary analysis of RCT	Of 2677 women enrolled at baseline, 1303 at 18-month f-up	Social behaviour intervention of Agriculture, livelihoods, male engagement, Caring for the Caregiver)	SBC + high cash reduced depression scores relative to standard of care, but no impact on proportion of women with depressive symptoms; relative to SoC, SBC + low cash & SBC alone had no impact on proportion of women with depressive symptoms; relative to SBC alone, adding cash reduced depression scores & proportion of women with depressive symptoms regardless of cash amount		Cash transfers integrated with SBC can benefit maternal PMH in rural Malawi during lean season.
Burgess et al. (2025); Ethiopia	Assess feasibility & acceptability of *Mother Time*	1-group, pre-post, mixed methods	16 postpartum women with mild-moderate depression & anxiety	3-session, small-group, community-health worker delivered CBT intervention addressing gap between MH challenges & postpartum FP use	feasible for CHW to learn & deliver intervention; able to use simplified materials to share MH information; challenge was limited time-CHWs would benefit from additional training, women likely benefit from additional sessions		Findings can be foundation for future research, pilot interventions to support all women to meet their PP & FP needs, including those with depression & anxiety
Callands et al. (2023); Liberia	Assess feasibility, acceptability, preliminary efficacy of CBT program on mental health	Cluster RCT	87 pregnant women aged 18-25	Project POWER: 10 session program x 5 weeks with concepts of mindfulness meditation, being present, acceptance, breathwork, coping control	PTSD symptoms decreased from baseline to 3-month assessment for all participants; intervention group had significant reduction in depressive symptoms		Intervention may be feasible & acceptable to promote mental & sexual health for young pregnant women; fully powered trials needed
Chibanda et al. (2014); Zimbabwe	Determine efficacy of group problem-solving therapy vs Rx drug therapy for PPD	RCT	58 PP mothers with PPD; problem-solving therapy: 30; drug Rx:28	*Friendship Bench*: 7 step plan with content on causes/ symptoms of depression, treatment options, assessment of suicide risk, involvement of appropriate family members in problem solving, support systems, follow-up	6 weeks post-intervention, drop in depression scores greater in the problem-solving compared to Rx amitriptyline group (P=.0097).		Intervention feasible & acceptable, more effective compared to drug Rx; could be integrated into MCH clinics to prevent mother-child HIV transmission
Cooper et al. (2002); South Africa	Can a community-based intervention address maternal depression & mother-child relationship	2-group convenience sample pilot study	32 pregnant women	Activities to encourage mother’s interactions to be responsive to infant’s individual capacities needs	Quality of mother-child engagement significantly more positive in intervention compared to comparison group; no impact on maternal mood		Preliminary evidence warrants formal evaluation of intervention
Cooper et al. (2009); South Africa	Assess efficacy of intervention to improve mother-infant relationship	RCT	449 pregnant women; intervention:220; comparison: 229		More infants of mothers in intervention than control group rated as securely attached at 18 months (P=0.029); depression scores for intervention group lower at both assessments than control group, treatment benefit only at six months (P=0.041); at 12 months (P=0.813)		If effects persist/are replicated, intervention holds promise for use in developing countries
Comrie-Thomson et al. (2022); Zimbabwe	Test intervention effectiveness on maternal MH at 0-6 months after childbirth	Cluster RCT	457 women with child <6 months of age & 242 partners; intervention:227; comparison: 230	*Mbereko+Men:* five 2-hour sessions, separate with women & men in gender-synchronized content, activities to improve men’s support for women & babies, coparents’ equitable, informed decision-making, maternal MH, care-seeking for MCH health services	Women’s mean depression scores declined in both arms with 34% greater decline in intervention arm (P = 0.008).		Low-intensity gender-specific intervention to improve maternal MH & care-seeking in setting of gender inequality
Dozio et al. (2020); Cameroon	Highlight impact of intervention on pregnant & lactating women & young children	Case study	1022 pregnant or lactating women (203 pregnant, 819 lactating) in 3 refugee camps	*Baby Friendly Spaces*: care for women during/after pregnancy with breastfeeding, psycho-social support, parenting skills, play sessions, child stimulation, relaxation, home visits	Women had improved wellbeing, reduced psychosocial suffering (p=0.000), increased perceived social support (p=0.000); positive maternal-child relations (p-0.000)		Results highlight accuracy of multisectoral approach in emergency context, focused on providing stable & protective environment
Eichenauer et al. (2023); Kenya	Determine if agricultural intervention affects stress, coping of pregnant women	Matched-pair, cluster RCT	1591pregnant women	Intervention households received 5 hermetic storage bags with capacity to store 100kg of maize, training in their use; compared to conventional polypropylene bags of comparison group	Pregnant women in treatment households perceived higher ability to cope with stress in 11 out of 12 months compared to those in the control households (p = 0.021).		Findings suggest use of hermetic bags gave pregnant women sense of empowerment to counter effects of food insecurity; research needed to examine long-term effects over several harvest seasons
Fink et al. (2024); Zambia	Test feasibility and acceptability of adapted intervention for distressed mothers in low-resource settings	Feasibility trial nested in cluster RCT	265 mothers with children <2 years old with mental distress; intervention: 134 comparison: 131	*Problem Management for Moms*: 10 one-hour weekly sessions of problem-solving, psychoeducation, cognitive restructuring, motivational interviewing, mindfulness exercises, effective communication	Intervention group had greater improvement in MH health scores post-intervention (p=.005)		Results suggest feasible & acceptable intervention; research needed to assess efficacy, suitability for integration into routine care
Green et al. (2020); Kenya	Gather preliminary data for PPD intervention for automated delivery via mobile phones	Plot, one-group case study, qualitative assessment	41 pregnant women, new mothers	*Healthy Moms*: automated, mobile phone delivery of *Thinking Healthy*, 15 sessions (3 prenatal, 12 postnatal) with 4-task format: 1) review key lessons from previous session, 2) review mood ratings, 3) teach skills, 4) practice-based homework	Most interviewees reported a positive attitude, attributed positive life changes to intervention; estimate that alpha version of Zuri led to a 7% improvement in mood	Results serve as baseline for future studies	
Gureje et al. (2022); Nigeria	Compare age-appropriate intervention of problem-solving therapy, behavioral activation, parenting skills to usual care	Cluster RCT	242 pregnant adolescents with depression; intervention: n=141; comparison: n=101	*RAPiD*: 6-session intervention of problem-solving therapy, behavioral activation, parenting skills training & support; “neighborhood mother” providing provision social support, parenting skills	Intervention group had lower level of depressive symptoms than control, (p = 0∙003) & better parenting practice (p = 0∙001)		Age-appropriate psychosocial intervention package holds promise for scaling up care for adolescents with PPD especially in resource-constrained settings.
Holmes et al. (2025); Ethiopia	Examine immediate & long-term effects of group CBT intervention	Cluster RCT	302 pregnant women	*Mothers Time:* 4 monthly sessions with 6-8 women using CBT techniques to reframe negative thoughts, build self-efficacy, develop problem-solving skills directly applied to FP decisions	Intervention clusters showed significantly greater reductions in symptoms for both depression & anxiety from baseline to follow-up & use of modern FP intervention compared to control clusters		FP services might consider PP mental health to support women’s reproductive intentions
Kaaya et al. (2022); Tanzania	Determine if stepped-care intervention by lay providers improves PP MH for women living with HIV	Cluster RCT	742 pregnant women with HIV intervention:395; comparison: 347	*Healthy Options:* stepped-care intervention of problem-solving therapy plus CBT for women with depressive symptoms at 6 weeks PP	Intervention had a 67% lower risk of PPD (p<0.001) at 6 months; at 9 months PP had 2% lower risk (p=0.281) corresponding to a 3.2%		Intervention had reductions in depression at 6 weeks but not 9 months PP; stepped-care may improve outcomes in early PP window
Kariuki et al. (2021); Kenya	Investigate effectiveness of brief, prophylactic/ therapeutic psychoeducational intervention	Cluster RCT	567 mothers; intervention:284; comparison: 283	10-25 minutes of PPD education, coping skills, healthy way of mother/child interaction & infant stimulation plus routine treatment	Depression score reduction among participants significantly associated with intervention (p = 0.040)		Brief intervention targeting mother & infant may reduce depression even in poorly resourced environments; can be integrated into existing MCH services.
Katzen et al. (2020); South Africa	Assess CHW efficacy in meeting Sustainable Development Goals in rural areas	Cluster randomized longitudinal cohort	470 new mothers assessed at 2 years after birth; SC N=313; HV N=157	*Philani Maternal, Child Health and Nutrition Trust* with CHWs receiving 4-week course on MCH followed by 2-week in-field training	Non-significant trend for mothers in HV group to show less depressive symptoms over time; alcohol use similar in both groups; HV mothers were more likely to report IPV than SC (p = .012)		CHW home visits resulted in better maternal caretaking, no direct benefits for infants in domains assessed
Katzen et al. (2021); South Africa	Assess 12-month outcomes of CHW program				HV mothers had significantly fewer depressive symptoms than mothers in SC; HV children attained a higher proportion of their developmental milestones; no other significant differences between the 2 groups		CHW programmes rapidly increasing, becoming critical components of task-shifting strategies of health departments in low- & middle-income countries
Kim et al. (2021); Kenya	Estimate impact of a cognitive-behavioral, group-intervention, on maternal depression & early childhood social-emotional development	Cluster RCT	417 pregnant women, mothers of children< age 2; intervention plus childhood development, n= 193; early child development only, n=224	*Integrated Mothers & Babies Course Early Childhood Development:* 14 in-person group sessions over 7 months with 5 booster sessions x 6 months with content to manage common PN stressors (mother-baby relationship, interpersonal/ social relationships, mood problems, unhealthy thought patterns)^2^	Intervention had no significant impact on reducing maternal depression or improving children’s socio-emotional development; sub-group analyses showed less depression in intervention women with no/low education, >4 children, no recent IPV experience of in the past year		Program may have potential to improve maternal mental health, early child development for more targeted vulnerable populations
Lasater et al. (2025); Ethiopia	Assess efficacy of CHWs in rural areas	Longitudinal prospective cohort study	470 mothers giving birth in district	Compared to mothers receiving SC, mothers who also received home visits by CHWs more likely to attend recommended 4 AP visits, exclusively breastfeed at 3 months, less likely to consult traditional healers at 3 months; mothers in both groups equally likely to secure child grant, have similar infant growth, 2-year developmental milestones	CHW training includes a four-week MCH, HIV & TB course, then 2 weeks of in-the-field training, shadowing of experienced CHWs; in field, CHW weigh children, provide health education about nutrition, breastfeeding, HIV, TB, AN care; file opened for all pregnant mothers & for under-weight, stunted or wasted children -who are regularly visited		CHW home visits resulted in better maternal caretaking, but did not have direct benefits for infants in assessed domains
le Roux et al. (2013); South Africa	Evaluate effect of CHW home visits in *Philani Intervention Program* on MCH from pregnancy through 6-months	Cluster RCT	SC: n=594; intervention (SC & CHW home visits) n=644	PIP condition received AN & PP home visits by CHW plus SC clinic visits; CHW had good social/ communication & problem-solving skills, 1-month training	Depression scores similar across both conditions		PIP is a model for countries facing significant reductions in HIV funding whose families face multiple health risks
le Roux et al. (2020); South Africa	Examine effect of CHWs in rural communities (*Philani Home-Visiting Model*)	Non-randomized 2-group comparison	1310 mother-infant pairs	CHWs focusing on maternal and child health, had been assigned to conduct home visits	Mothers with home visiting reported fewer depressive symptoms, attended more AN visits, better baby-feeding practices		Impact of CHWs in rural area less pronounced than in peri-urban areas; CHWs likely to need enhanced support & supervision in rural context
Lund et al. (2020); South Africa	Determine effectiveness of task- sharing psychological treatment for PPD using CHWs	RCT	384 pregnant women scoring >13 on EPDS; intervention:184; comparison: 200	Structured 6-session psychological treatment with content to promote resilience, cope with life circumstances, problem solving, psychoeducation, behavioral activation, healthy thinking, birth preparation, relaxation	No significant differences in HDRS scores (primary outcome) from baseline to 3- or 12-months PP; significant change in mean EPDS scores from baseline to 3 months PP significantly greater in intervention compared to control group		Task-sharing treatment not effective in treating PPD; findings give cause for reflection on strategy of task-sharing in low-resource settings
McKinnon et al. (2020); Senegal	Can model of group AN care address challenges of pregnancy care & education	Effectiveness–implementation RCT	378 pregnant women; intervention:245; comparison: 85	*Group-ANC*: structured 2-hour group sessions of AN care group education activities, peer support	As part of positive maternal health outcome, depression scores decreased, but not significant;		Promising model to promote positive experience during AN care & childbirth
Mhango et al. (2024); Malawi	Assess feasibility, acceptability & impact of MH intervention for pregnant adolescents	One-group cohort study with mixed methods evaluation	19 pregnant adolescents	*FOR MAMA:* 5 individual & group session psychosocial intervention delivered by CHWs, focused on problem solving, coping, relationships, mitigating strategies^1^	Significant improvements (p<0.05) on 2 measures of general mental health; lateness to sessions, community attitudes were feasibility challenges		Intervention proved an acceptable & feasible psychosocial intervention for pregnant adolescents
Mhango et al. (2025); Malawi	Gain insight into stakeholders’ preferences for scalable intervention for common mental problems among PN adolescents	Participatory qualitative research	10 PN adolescents, 8 family members, 10 HC providers		Emphasized need for information on causes, symptoms of common MH problems, developing coping strategies focused on external stressors—problem-solving, financial literacy and interpersonal skills—& on emotion-focused approach behaviours—behavioral activation, relaxation and anger management		Findings informed design of a brief multicomponent guided intervention for adolescents in the antenatal period
Murnane et al. (2025); Kenya	Examine if effects of agricultural intervention on food insecurity & psychosocial outcomes remained robust in pregnant compared to non-pregnant women living with HIV	Secondary analysis of cluster RCT	396 women, 370 who became pregnant	Loan to purchase human-powered irrigation pump, fertilizer, seeds & pesticides, plus provision of training in sustainable agriculture & financial literacy	In comparison to controls, intervention associated with greater decline in food insecurity among women who did & did not became pregnant; comparable psychosocial outcomes in pregnant and non-pregnant women		Agricultural livelihood interventions may hold promise for improving pregnancy outcomes through improved maternal food security
Mutisya et al. (2018); Kenya	Assess effect of psychosocial intervention on GBV, depressive symptoms	Cluster RCT	288 pregnant women with positive GBV score	3 30-minute sessions providing information on meaning & magnitude of GBV, GBV cycle, adverse effects during pregnancy, safety assessment, identification of safety measures^3^	Intervention group had significantly lower mean depression scores compared to the usual care post-intervention, (p < 0.0010)		Intervention aimed at secondary prevention of GBV, improvement of mental health in pregnant women holds promise
Nakku et al. (2021); Uganda	Evaluate impact of MH intervention delivered by non-specialist health workers in women with PPD	One group with assessments at baseline, 3, 6 months	153 pregnant women with depression	*Programme for Improving Mental Health Care:* training midwives to assess, diagnosis, treat PPD with evidence-based treatment, group-based problem-solving therapy, interpersonal violence support; antidepressant medication	34.7% of women received >4 group PST sessions; significant reduction in depression scores (p < 0.001) at midline & endline		Intervention in primary care by trained midwives in real-world setting may lead to improved outcomes for women with PPD
Ng’oma et al. (2024); Malawi	Test acceptability & feasibility of PPD psychosocial intervention	One group, pre-post- test, mixed method evaluation	24 pregnant women with EPDS scores >12	*Thinking Healthy Programme–Peer Delivered:* family focused 8-session adaptation of THP, simplified for use by peer volunteers; developing relationships, psychoeducation, family support, behavioral activation, problem solving	Significant difference between pre- post-test EPDS scores one week after 8th session (p<0.001);		Intervention may be useful in management of PN depression in primary care settings; definitive trials needed to evaluate effectiveness
Notiar et al. (2021); Kenya	Test feasibility & acceptability of group psychosocial intervention plus culturally adapted CBT	One-group, mixed method feasibility study	Depressed mothers with children aged 0-36 months	*Learning Through Play:* 12-session integration of 2 interventions-includes supportive & mponents, problem-solving techniques to address maternal depression & child-related difficulties, parenting program to promote early child development	High feasibility& acceptability; depression scores decreased at 12 weeks post baseline(p<.001); four themes: perceived positive outcomes from help-seeking behavior; socio-economic factors as driver of depression; perceptions of program usefulness; lack of access to care		Results indicate intervention is feasible & acceptable; need to study clinical outcomes & cost-effectiveness in larger RCT, with longer follow-up period
Patil et al. (2025); Malawi	Test Centering-based group AN model	Hybrid effectiveness RCT implementation trial	1887 pregnant women; intervention: group AN care, n=946; control: SC, n=941	Group AN care theory of change model^4^ of were 2-hour AN, 1 PP contacts; Centering-based pattern= first 30–45 minutes socializing/ health assessment, one-on-one health assessment with midwife	Group AN care group reported less mental distress in late pregnancy		Study enhances understanding of group AN diverse positive impact
Poku et al. (2022); Botswana	Develop & assess effectiveness of a tailored anti-stigma intervention for pregnant women living with HIV	Two-group pragmatic design to develop content	59 HIV positive pregnant women; intervention: 49 comparison: 10	*Mothers Moving Toward Empowerment:* 8 weekly 90-minute sessions with themes of respected motherhood & cultural components that protect against stigma^5^	Significant decreases in HIV-related stigma and depressive symptoms at four-months PP compared with control group		Intervention initially effective in targeting culturally salient aspects of HIV stigma
Rotheram-Borus, Tomlinson, et al. (2014); South Africa	Examine MCH outcomes from CHW home visits	Cluster RCT	Control: clinic care, n=594; Intervention: clinic care & home visits by CHWs, n=644	*Philai Maternal Child Health and Nutrition Programme* trained CHWs to implement home visits; CHWs selected from community role models, trained in basic strategies to change thoughts, feelings, behaviours, targeted specific local health challenges	On 7 of 32 outcomes, significant benefits for intervention compared to control (p = 0.008); benefits related to number of home visits; no significant difference in depression scores		Home visits to by CHWs may be a feasible strategy for enhancing MCH outcomes; visits likely must extend over several years for persistent benefits
Rotheram-Borus, Richter, et al. (2014); South Africa	Evaluate effect of clinic-based support by HIV-positive Peer Mentors among Women Living with HIV	Cluster RCT	Standard care, n=656; intervention: standard care & support by HIV positive peer mentors, n=544	8 meetings with peer mentors	Intervention group women living with AIDS had significantly fewer depressive symptoms & underweight infants than control group		Women living with HIV benefit by support from HIV-positive Peer Mentors, even though participation was partial, with incomplete follow-up rates
Rotheram-Borus et al. (2023); South Africa	Examine maternal outcomes at 5- and 8-years’ post-birth, from *Philani Intervention Program*	Follow up of RCT	Standard care, n=430; intervention, n=472	SC=clinical services + lifelong antiretroviral therapy for mothers living with HIV (MLH); intervention=clinical services & PP home visits by CHWs designed to promote foundational skills, awareness of feelings, key messages plus brief intervention on alcohol prevention	Intervention mothers reduced depression versus standard care at 3 years disappeared by 5 & 8 years; depression prevalence declined from 35.1% prenatally to 5.5% at 8 years, fewer intervention mothers drank alcohol and reported problematic use at 5 but not 8 years; prevalence of HIV+ did not differ by condition, increased from 26% - 45%		Dissipation of early child benefits from home visiting by age 8 years likely reflects lack of durable change in maternal behaviors plus social-cultural factors, cumulative effects of community deprivation; high AN rates warrant screening & treatment for depression in standard AN care; low-middle income countries may need sustained interventions, plus alcohol use reduction, to capitalize on initial gains from interventions & address community level factors
Sanfilippo et al. (2020); The Gambia	Examine feasibility of musical engagement intervention to reduce common mental disorders in pregnant women	Cluster stepped-wedge RCT	124 women 14-24 weeks pregnant; intervention: 50; comparison: 74	*CHIME:* 6 weekly 1-hour music-based sessions psychosocial support sessions designed to lift mood, build social connection, provide health messaging	At 4 weeks, intervention compared to control group had mean reduction on two instruments (p<0.01); 72% attendance at intervention of >3 sessions; intervention acceptable		Trial able to address main objectives, showed feasibility, acceptability, and potential benefit compared with standard care
Saronga et al. (2024); Tanzania	Estimate cost-effectiveness of intervention in managing PPD in women with HIV	Two-group post-effectiveness analysis of Kaaya et al. (2022)	742 women with HIV infection; intervention: 395	*Healthy Options*: 1 individual & 6 group psychosocial support session intervention with problem-solving & cognitive behavioral therapies facilitated by CHWs	Statistically significant decrease in mean depression score between intervention/control arms at 6 weeks (p<0.01) & 9 months follow-up (p<.05)		Analysis did not capture all benefits & costs; CHWs a less costly alternative to MH professionals
Seward et al. (2025); South Africa	Estimate indirect effects of mediators through which the intervention reduced PN depressive symptoms	RCT	310 women <28 weeks pregnant; intervention: 136 comparison: 174	*AFFIRM-SA*: 6 weekly sessions delivered by CHWs to reduce symptoms of PPD in context of social, economic, interpersonal adversity, including problem solving skills, behavioral activation, healthy thinking, relaxation training, birth preparation	Effect of intervention measured through EPDS score differences which improved by >40% between treatment arms, 8% mediated through reduced levels of food insecurity, 9% through increased levels of perceived social support; no evidence to support mediating role of reduced violence		Adding psychological components with content that directly target social determinants of mental health (such as food insecurity, social support) could improve PPD
Sigwebela et al. (2026); South Africa	Develop Community Health Intervention through Music Engagement to support OMH	CBPR with 5 focus groups:	CHWs, music experts, traditional healers, health care workers, health NGO management	Build on existing musical practice of CHW, by the approach co-developed in The Gambia	4 themes: challenging to PMH; existing community music practices; benefits of music making in supporting social connections; factors important for development of music-making intervention to support PMH		Findings suggest potential for implementing music-based MH interventions in South Africa, adaptable to various facilitators & community contexts
Spedding et al. (2020); South Africa	Investigate task sharing problem-solving therapy intervention; evaluate feasibility & acceptability	One-group pre-post feasibility study	38 pregnant women scoring >15 on EPDS	3 sessions with trained counselor using problem solving therapy	Significant reductions on 2 PPD instruments; acceptability lay in opportunity for confidential disclosure of problems, relieving staff of burden of managing patient distress; barriers were lack of transport, work commitments		Results support task sharing to treat common mental problems in PN primary health care settings; research needed on how to integrate programs into public health settings
Spelke et al. (2022); Zambia	Assess feasibility of trial of interpersonal psychotherapy vs. antidepressant medication to treat PPD & anxiety in PP women with HIV	RCT feasibility trial	80 PP HIV positive women with diagnosis of major depression or generalized anxiety	Consenting participants were randomized 1:1 to up to 11 sessions of interpersonal therapy or daily self-administered sertraline	Visit adherence greater in medication arm (p = 0.06); depression scores decreased in both groups with mean change greater in therapy group (p = 0.04); both groups had similar changes in mean log viral load		2 forms of PPD treatment feasible and acceptable among women with HIV; both improved depression; full-scale trial required to determine if treatment improves maternal–infant HIV outcomes
Tessema et al. (2025); Ethiopia	Assess effectiveness of antenatal group psychoeducation in improving PPD literacy	Cluster RCT	550 antepartum women from 32 health centers (16 per arm)	5 antenatal group-based psycho-education sessions on PPD knowledge, prevention, therapy, with content from validated guidelines, including WHO guide	Significant difference between groups in overall mean PPD literacy score, ability to recognize PPD, knowledge of risk factors & causes, access to PPD information (p = 0.001)		Antenatal group psycho-education had moderate effect on overall PPD literacy; further research needed to effectively change negative help seeking attitudes
Tomlinson et al. (2016); South Africa	Examine maternal/ child outcomes of CHW home visiting program at 36 months	Cluster RCT	1248 pregnant women; intervention:654; comparison: 594	*Philani Intervention Program:* home visits for baby’s first 3 years by CHWs trained to address multiple health challenges	Compared to comparison group @ 36 months post-birth intervention mothers significantly less depressed, children significantly likely to be hospitalized or stunted, had better vocabularies, less depression on 2 measures (p = 0.009; p=0.011)		CHW approach grounded in cognitive-behavioral skills, with local-tailored content addressing local health risks, supervision, accountability
Wagner et al. (2023); Uganda	Assess if depression care model affects improvement in HIV care adherence, early child development, maternal functioning	Cluster RCT	354 pregnant women living with HIV with mild depressive symptoms; intervention:177; comparison: 177	*M-DEPTH*: depression screening, psychoeducation, treatment recommendation (Problem Solving Therapy, for moderate, drug Rx for severe depression); 7 biweekly individual treatment sessions	Strong intervention effect on maternal depression at post-partum visits assessment; moderate effects on depression on maternal functioning (self-care & infant care); minimal effect on depression reduction on early child development, maternal viral suppression, or ART adherence		Depression care for pregnant women with HIV important for mental health, helps women better manage parenting/infant care; extra interventions needed for early child development
Washio et al. (2024); South Africa	Pilot test 3-month incentive-based text-messaging intervention targeting maternal alcohol use during pregnancy & lactation	One-group pilot study	60 participants, 31 pregnant, 29 lactating	*Contingency management*: alcohol abstinence confirmed by urinalysis twice weekly & weekly health-related text messaging from evidence-based brief intervention	Days with >4 drinks in past month decreased from baseline 9 days to 1–3 days follow-up (p-values: 0.144–0.010); significant increases in participants with alcohol-negative urine tests (p < 0.001); no decrease in mean depression scores (p=0.546)		Further testing warranted in well-powered RCT
Woollett et al. (2025); South Africa	Describe rationale, approach, feasibility, mechanism of change of art-based intervention	One-group pre-post study	28 pregnant/ parenting adolescents with IPV	6-session arts-based psychoeducation delivered by 4 graduate art therapy students with clinical supervision; content of collage, body mapping, projective drawing, sand tray with psychoeducation, mindfulness practice, safe parenting skills	Pros: new socio-emotional skills; mutual support of reduced loneliness; skilled, trustworthy facilitation; positive future outlook; Cons: concern re confidentiality; complexity of art materials; 23% loss to follow-up		Findings confirm therapeutic advantage of integrating non-verbal methods in treating adolescent MH
Yang et al. (2022); Botswana	Assess whether targeting intersectional stigma more effective than addressing HIV-related stigma alone	Pragmatic design, with pre-post data at baseline, 4 months PP	59 pregnant women with HIV infection; intervention:44 comparison:15	*Moving Mothers toward Empowerment*: 8-group sessions, led by trained counselor & peer mother with HIV, of based strategies to reduce stigma, counter intersectional stigma by promoting capabilities to achieve “respected motherhood^”6^	Intervention group had significant decreases in HIV stigma & depressive symptoms from baseline to 4-montths		Results a significant step forward to address intersections of multiple systems of oppression
Yator, John-Stewart, et al. (2022); Kenya	Examine program feasibility, acceptability & effectiveness improving depression & ART adherence	2-group RCT with intention to treat wait list, mixed methods evaluation	24 mothers (ages 18-24) 6-12 weeks PP living with HIV scoring >10 on EPDS	*Interpersonal Therapy-Group*: 8 weekly sessions of a brief psychotherapy for depressed adolescents that addresses developmental tasks, clinical aspects of depression, treatment barriers, ^7,8^	-Intervention group had significant decrease in depression scores compared to control group (p 0.017); CHWs found intervention useful for their own knowledge; 21/24 adolescents attended all sessions; primary health workers embraced intervention, accommodating sessions into clinic activities; themes: improved communication & anger management, lessening of HIV-related stigma		Provides preliminary evidence that IPT-G delivered by CHW can have benefits for PPD among young mothers with HIV
Yator, Khasakhala, et al. (2022); Kenya	Assess acceptability & impact program delivered by CHWs to depressed PP adolescents living with HIV						

^1^Lazarus and Folkman’s transactional stress theory; ^2^Attachment theory; ^3^ Dutton’s empowerment model; ^4^ Group AN care theory of change model^; 5^Stigma; What Matters Most theory; ^6^ Intersectional stigma; ^7^ Task-sharing implementation strategy; ^8^ Capability, Opportunity, Motivation model

**Abbreviations** AN-antenatal, ART: Antiretroviral therapy, BFH: Baby Friendly Hospital, CBPR: community-based participatory research, CBT: cognitive behavioral therapy, CHW: community health worker, EPDS: Edinburgh Postnatal Depression Scale, FP: family planning, GBV: gender-based violence, HDRS: Hamilton Depression Rating Scale, HIV: Human Immunodeficiency Virus, HM: Home visit, IPT-G: Interpersonal Psychotherapy Group, IPV: interpersonal violence, MCH: maternal-child health, M-DEPTH: Maternal Depression Treatment in HIV, MH: mental health, NGO: non-government agency, PP: postpartum, PPD: perinatal/postpartum depression, PMH: perinatal mental health, PN: perinatal, PTSD: posttraumatic stress syndrome, RAPiD: Responding to the challenge of Perinatal Adolescent Depression, RTC: randomized-control trial, SC: standard care, WHO: World Health Organization

## Results

Between 2002 and March 2026, 57 studies were identified, originating in 18 countries of SSA. Sixteen were conducted in South Africa [31–46] and nine in Kenya [47–55]. Six studies were conducted in Malawi [56–61], and five in Ethiopia [62–66]. Two studies in Zimbabwe [67,68] and three in Tanzania [69–71], and two each in Zambia [72,73], Uganda [74,75], and Botswana [76,77]. The countries of the Democratic Republic of Congo [78], Sierra Leone [79], Angola [80], Ghana [81], Liberia [82], Cameroon [83], Nigeria [84], Senegal [85] and The Gambia [86] each had one study, while Ghana and Uganda had one study conducted in their two countries [87].

### Research designs

Several studies used strong designs of randomized control trials [37,40,42,43,52,54–56,59,60,64,67,73,79,85]. More common, and equally strong was the use of cluster-randomized control trials [31–33,35,36,45,47,48,50–52,62,63,68–70,72,75,78,80–82,84].

Four studies use a two-group pre-post designs [34,41,76,77], generally to assess feasibility and acceptability and calculate preliminary results as the basis for a future full-scale study.

Some studies used a one-group, pre-post quasi-experimental design [39,44,46,49,53,57,58,65,74,87], while one study used a one-group longitudinal design [66].

One study conducted a cost-effectiveness assessment with a comparison group [71], one used case study methods [83], and two used qualitative methods [38,61].

Table 4 summarizes the number of included studies by country and by research design. Cluster-randomized controlled trials were the most common approach (24 studies, 42%), followed by individual RCTs (14 studies, 25%) and non-randomized quasi-experimental pre-post designs (13 studies, 23%); qualitative, cohort, mixed-methods, and case study designs together accounted for the remaining six studies. Study representation by country largely mirrored the concentration of maternal mental health research in the region, with South Africa (16 studies), Kenya (9 studies), and Malawi (6 studies) together accounting for more than half of the evidence base, while nine countries were represented by only a single study each.

**Table 4:** Number of included studies by country and research design (N = 57).

Category	n
**Country**	
South Africa	16
Kenya	9
Malawi	6
Ethiopia	5
Tanzania	3
Zimbabwe	2
Zambia	2
Uganda	2
Botswana	2
Democratic Republic of Congo	1
Sierra Leone	1
Angola	1
Ghana	1
Liberia	1
Cameroon	1
Nigeria	1
Senegal	1
The Gambia	1
Ghana & Uganda (joint, multi-country study)	1
**Research Design**	
Cluster RCT (incl. cluster-randomized cohort, matched-pair, and stepped-wedge designs)	24
Individual RCT (incl. waitlist, pilot, and effectiveness–implementation RCTs)	14
Quasi-experimental / non-randomized pre-post design	13
Qualitative (incl. community-based participatory research/focus groups)	3
Longitudinal cohort study	1
Mixed methods (general)	1
Case study	1

*Totals for each section adds up to 57, matching the number of studies listed in* Table 3*. One study (Bam et al., 2026) was conducted jointly in Ghana and Uganda and is counted once under the combined heading rather than separately under each country.*

### Participants/Samples

Most studies focused samples of pregnant, postpartum, lactating women or women with young children. Seven studies enrolled mother-young child pairs [34,51,53,68,69,72,78] and assessed both mother and child outcomes. The study by Comrie-Thomson, Webb (68) also included women’s partners.

Mothers with concerning mental health or PPD scores were the focus of twelve studies [42,44,53–55,58,64,65,67,72,74,84] (Notiar, et al., 2020; Speeding, et al., 2020; Yator, et al., 2021 and 2022). The samples of Spelke, Paul (73) and Wagner, Gwokyalya (75) were made up of women who were both HIV positive and had a mental health diagnosis.

Specific populations were the focus of nine studies which included only HIV positive pregnant women [35,36,54,55,70,71,73,75–77] and five which included pregnant adolescents or women less than 25 years of age [39,57,61,82,84]. One study worked with pregnant women with positive gender-based violence scores [52]. Bam, Diji (87) worked with health professions students while Sigwebela, McConnell (38) worked with traditional healers and health care workers.

The studies with cluster randomized designs had the largest sample scope and size, ranging from 87 young pregnant women [82] to 9039 women receiving perinatal care from 190 clinics in one providence of Angola [80]. The studies characterized as pilots generally had small sample sizes, ranging from the ten participants in the study by Mhango, Michelson (61) to the 124 in the study by Sanfilippo, McConnell (86). One article that used a case study approach was conducted in three refugee camps [83] with 1022 participants.

### Intervention content

Virtually all educational programs delivered their content in at least some group sessions. General postpartum education and support, as well as development of coping skills made up the intervention of several articles [49,54,55,57,58,63,72,76,79]. Three author teams combined this general approach with a focus on the mother-child relationship [50], a problem-solving approach [42], and engagement [86]; one author team evaluated music engagement along [38] and one focused on arts-based psychoeducation [39]. Five articles focused on mother-baby relationships [40,41,69,81,83]. Seven interventions used problem-solving therapy as a focus [44,56,64,67,75,84] or with other components such as CBT [70]. Seven studies used techniques of CBT [43,51,53,62,71,81,82]. Medication to address PPD was offered as an intervention arm in two studies [67,73].

In other than specific PPD content, community support for breastfeeding was offered and evaluated, with PPD as a secondary outcome [78]. Group antepartum care was evaluated in two studies [60,85]. Increasing knowledge about and strategies to address GBV was content evaluated by Mutisya, Ngure (52). Other intervention topics included gender-specific activities to improve men’s support for women and babies [68], contingency management for alcohol use [46], addressing HIV-related stigma [77] and providing medication and support for mothers with HIV infection [35,37]. Seven studies evaluated home visits by community health workers [31,33,34,36,45,65,66].

Moving beyond the individual and the group level, only two studies focused on changed in clinical practices, one with the distribution of a Maternal-child health (MCH) Handbook with protocols and training for perinatal providers, as well as community education [80], and a second assessing an interactive professional education program on perinatal mental health (PMH) [87].

Two studies focused on possible socio-economic focus factors as a mechanism for influencing PPD. Eichenauer, Huss (48) assessed a simple strategy to see if storage bags that preserved food and countered food insecurity could address stress and coping. One of the three arms of the study by Bliznashka, Yousafzai (69) included a cash transfer along with visits with community health workers. Cash transfers during the agricultural lean season and loans to purchase agricultural supplies were concrete strategies of two studies [47,59].

Only two studies used technology in the form of regular cell phone contact for intervention delivery or text messaging [46,49]. In contrast, nine studies used trained community women (knowns variously as community health workers, lay health workers, trusted mothers) to delivery intervention content [43,45,57,58,69,77,79,81,84].

Seven studies specified a theoretical basis for their interventions. Agler, Zivich (78) used transactional stress theory as the important difference in including Step 10 of the Baby Friendly Hospital Initiative. The work of Kim, Opiyo (51) used attachment theory as the basis of their work which focused on mother-baby relationships. Mutisya, Ngure (52) used an empowerment model with participants. Both Poku, Becker (76) and Yang, Eschliman (77) based intervention content on stigma, while Yator, Khasakhala (55) and Yator, John-Stewart (54) incorporated task-sharing and a capability, opportunity and motivation models.

### Study outcomes

The majority of studies had positive mental health results for participants, either as a primary or secondary outcome.

Six studies which used cognitive behavioral therapy (CBT) as a primary method all showed significant reductions in depressive symptoms from baseline to follow-up assessments [43,51,53,62,71,82]. Of note is that the sub-group analysis of Kim, Opiyo (51) had demonstrated strong effect on women with no/low education, four or more children, and/or no recent IPV experience of in the past year. Only one study using CBT [81] found no significant difference between intervention and comparison groups at eight-month follow-up; however, PPD rates were only 3% in both groups.

The four studies that used problem-solving therapy as their main intervention strategy all showed positive results in feasibility and acceptability [64], remission of depression and retention in HIV care [56], in feasibility, acceptability and managing distress [44], and in maternal depression at postpartum visits [75]. When combined with CBT, problem-solving therapy showed a 67% lower risk of PPD at six months postpartum [70]; when combined with a peer-led group session, PPD was significantly lower than in a comparison group [79]. Positive results were also seen in interventions where problem-solving was used in combination with a variety of other modalities [67,84].

The two studies using medication to address PPD as an intervention arm found it to be less effective in comparison to problem-solving in one [67] and to interpersonal therapy in a second [73].

Some results were a bit difficult to interpret. The CHW-delivered psychological intervention showed no between group changes with the Depression Rating Scale, the primary outcome) but significant changes with a second instrument (Edinburgh Postnatal Depression Scale, a secondary outcome) between baseline and three months post-intervention.

The pilot study of Cooper, Landman (41), with its focus on mothers’ responsiveness to infant’s needs had no impact on maternal mood; however, the larger RCT [40] reported significant between-group differences at six months.

Three studies showed no significant change or between-group differences in mothers’ depression scores. The studies with intervention content on mother-child bonding and child development, included both mother and child findings. Baumgartner, Ali (81) found no significant effects on mental health or child development as a result of their multi-session work delivered by lay-counsellors. The pilot study of Cooper, Landman (41) found mother-child engagement was more positive in the intervention than the comparison group while there was no effect on mother’s mental health. In the larger RCT of Cooper, Tomlinson (40), mother-child had positive attachment at 18 months compared to the comparison group, while depression scores were significantly more positive at six but not at twelve months. The work of Dozio, Le Roch (83) found significantly positive pre-post changes in mothers’ psychosocial and social support assessments, as well as in maternal-child relationships. The community health worker intervention of Bliznashka, Yousafzai (69) significantly reduced depression scores compared with control and had positive effects on maternal responsiveness, and some positive effects on maternal-infant responsiveness.

Other community health worker interventions had results that were difficult to interpret, with five evaluating some facet of the *Philani Intervention Program*. The work of both Tomlinson, Rotheram-Borus (45) and Katzen, le Roux (32) found intervention mothers to be significantly less depressed, while Rotheram-Borus, Tomlinson (36) and le Roux, Tomlinson (33) found no significant difference in depression scores between intervention and control groups.

Minimal outcome differences were found in the studies which assessed changes in care through group AN care [60,81] or as the result of MCH handbook distributed to clinics and throughout communities. The training of health professionals on PMH was found to have found to have high feasibility and applicability among participants [87].

The four interventions that addressed economics had different results. Bliznashka, Yousafzai (69) intervention arm that included a cash transfer along with visits with community health workers to address PPD showed no statistical difference from comparison arms; however, the follow-up results of this intervention found that adding cash did result in reduced depression scores [59]. But Eichenauer, Huss (48) focus on a strategy to address food insecurity found positive results in women’s sense of empowerment and ability to cope with stress, and Murnane, Bukusi (47) intervention of agricultural supplies had no effect on women’s psychosocial outcomes.

## Discussion

This scoping review set out to map interventions addressing maternal mental health broadly, including perinatal depression, anxiety, post-traumatic stress disorder, and suicidal ideation, across Sub-Saharan Africa. Of the 57 eligible studies identified across 18 countries, however, every intervention addressed postpartum depression as a primary or secondary outcome; no eligible studies evaluated interventions targeting anxiety, PTSD, or suicidal ideation specifically. This concentration of the evidence base on PPD, rather than the full spectrum of maternal mental health conditions named in the Introduction, is itself a notable finding: it suggests either that intervention research in SSA has disproportionately prioritized PPD relative to other maternal mental health conditions, or that literature on anxiety-, PTSD-, and suicidality-focused interventions exists but falls outside our English-language, peer-reviewed, database-indexed search parameters. While SSA covers a vast area, the number of studies identified in the review that address the mental health problem of PPD is encouraging. The interventions in the studies were delivered across 18 countries, used research methods, assessed a range of content and delivery strategies, and yielded largely positive results. Reflecting this concentration on PPD, the 57 included studies spanned a range of rigorous designs, predominantly cluster-randomized and individual randomized controlled trials, alongside quasi-experimental, qualitative, and case study approaches (Table 4), and evaluated interventions ranging from individual psychotherapy and problem-solving therapy to community health worker-delivered programs, economic interventions, and technology-based tools, the majority of which reported positive effects on maternal depressive symptoms or related psychosocial outcomes.

The majority of studies used mixed interventions of cognitive behavior therapy and problem-solving therapies and had largely positive results, even when delivered by trained non-professionals. They reflect the state of the science about the strong potential for the use of these strategies [88]. The results are encouraging and suggest a robust will to address the mental health problem of PPD. They also demonstrate that identification and treatment of PPD is acceptable to local populations, and potentially feasible for implementation. However, the interventions all face scale-up challenges, including financing, ongoing recruitment, training and supervision of health workers, and the need for mechanisms to ensure fidelity [89,90]. No studies were identified which addressed these challenges, suggesting a serious gap in moving forward with significant resource allocation.

These findings extend a substantial global evidence base showing that non-specialist, task-shared psychological and psychosocial interventions can meaningfully reduce perinatal depressive symptoms. A landmark cluster-randomized trial in rural Pakistan demonstrated that a CBT-based intervention delivered by Lady Health Workers nearly halved the risk of perinatal depression among mothers, a finding that informed the World Health Organization’s subsequent global endorsement of the Thinking Healthy Program for use in low-resource settings [91]. A systematic review of psychological interventions delivered by non-specialist health workers across low- and middle-income countries similarly found that effective programs shared several features present in the SSA interventions reviewed here: integration within routine maternal and child health services beginning antenatally, a focus extending beyond the mother to the child and family, and attention to the social determinants of distress alongside individual psychological content [92]. Set against the Cochrane review of psychosocial and psychological interventions for preventing postpartum depression, drawn predominantly from high-income settings including Australia, the United Kingdom, the United States, and Canada, the SSA evidence synthesized here suggests that comparable effects can be achieved when care is task-shifted to trained lay health workers rather than restricted to professional providers [93], reinforcing task-sharing as a viable strategy for closing the perinatal mental health treatment gap in resource-constrained settings [18].

Comparison with our team’s parallel scoping review of postpartum depression interventions in South Asia [27] is also instructive. That review identified 20 eligible studies across only four of eight South Asian countries searched, similarly delivered predominantly by community health workers using culturally adapted psychosocial content, and reached a comparable conclusion: the evidence supporting effective PPD interventions already exists, and the more pressing gap lies in political and policy commitment to scale delivery rather than in identifying what works. The present review’s larger evidence base, 57 studies across 18 of 48 SSA countries, more than twice the country coverage found in South Asia, suggests SSA-based intervention research is comparatively more mature in scope, yet faces the same fundamental translation gap from efficacy to system-level implementation.

The focus of the PPD interventions that focused on strengthening the mother-baby connection [34,40,41,69,81,83] make initiative sense because of the negative effects on infant development as a result of inadequate bonding. Two of these studies [41,81] showed no positive effect on mothers’ moods; however, Cooper, Tomlinson (40) larger scale study did have a significant improvement in the intervention group’s depression scores when compared to the comparison group. The decision by researchers on intervention content is guided by experience with both the program and the population. For example, pediatricians, while appreciating the maternal stress of PPD, may be more professionally inclined to assess content that focuses on infant content and outcomes. However, peer-reviewed literature can also serve as a guide, in this case with a recent finding that a higher proportion of mother-focused interventions [20/30 (66.7%)] brought significant reduction in PPD outcomes as compared to a lower proportion of mother-child focused interventions [4/7 (57.14%) [94]. The evidence favors mother-focused interventions for improving PPD with mixed interventions being more effective [94]. The use of such results in the development of future interventions is recommended.

The four studies in this review that focused on possible socio-economic factors as an intervention for PPD [47,48,59,69] had a basis in the published literature. A systematic review from Turkey found economic status to be a strong predictor of PPD [95]. Economic stress was a predictor in a meta-analysis focusing on Latinas in the United States [96] and poor economics a factor in an analysis of PPD risk factors in the Middle East [97]. Finally, in a review of PPD etiology on African countries, women with lower economic status had a statistically higher risk of PPD [6].

The use of technology in the form of regular cell phone contact was found in only two studies [46,49]. This would seem to be a missed opportunity in light of the conclusions of a scoping review on PPD in low-and-middle income countries suggesting the high potential for technology-based interventions such as mobile phone applications. The success of such mHealth interventions is, of course, a function of the widespread availability of cell phones, charging and data; researchers and outcomes from their work will always be a bit behind this massive communication transformation [98].

The unique approach of Sanfilippo, McConnell (86) of using music therapy was mirrored in the positive effects found a recent meta-analysis whose results suggested the effectiveness of this modality in low-and-middle income countries [99].

The two studies in which anti-depressant medication was a comparison arm [67,73] were published or conducted before a Cochrane review article found limited evidence regarding the effectiveness and safety of antidepressants in the management of postnatal depression, particularly for those with more severe depression [100]. The role of stigma in the use of medication for PPD treatment suggests this intervention supports its minimal use as an intervention in future studies [101].

Interventions that focus on the health system, clinic, community or provider education level have the potential for greater impact than those working with individual women. Only one intervention identified in this review [80], worked to change clinical practices with the distribution of the MCH Handbook to document all mother and child health services and conditions into a single document. While PPD rates were just one of several secondary outcomes, the primary outcome of continuity of care was not changed. Furthermore, no research was identified which focused on strengthening midwifery training curricula to include the diagnosis of and appropriate treatment for PPD. Since many midwives and nurses working with pregnant and postpartum women cannot identify and manage PPD, strengthening basic curricula and providing continuing education programs could result in an overall improvement of care [102,103]. A recent systematic review found such significant benefits with educational courses offered in a variety of formats [104]. Finally, no interventions were identified at any level, individual, professional, or clinical, which assessed the cost-effectiveness of addressing PPD, suggesting the difficulty in assessing women’s economic contribution to family and community in many areas of SSA.

### Limitations

Consistent with scoping review methodology, we did not conduct a meta-analysis; even among topically similar subsets of included studies, such as the six studies using cognitive behavioral therapy as a primary intervention, marked heterogeneity in comparator conditions, outcome measures and instruments (e.g., the Edinburgh Postnatal Depression Scale versus the Hamilton Depression Rating Scale), and follow-up timepoints precluded valid quantitative pooling of effect estimates. Several further limitations should be considered when interpreting these findings. First, the search was restricted to English-language publications; given that several SSA countries are predominantly Francophone or Lusophone, relevant intervention studies published in French, Portuguese, or other languages may have been missed, potentially underrepresenting maternal mental health research conducted in these regions. Second, consistent with scoping review conventions, grey literature, including NGO program evaluations, government reports, and unpublished dissertations, was excluded; because many maternal mental health interventions in SSA are implemented and evaluated outside academic publishing pipelines, this may have omitted relevant programmatic evidence. Third, initial screening and study selection were conducted primarily by one author, with a second author consulted only where inclusion was uncertain, rather than through fully independent dual screening of all records; while consensus-based resolution was used throughout, this design carries a somewhat higher risk of missed or inappropriately excluded studies than a dual-independent-screening approach. Finally, as is standard for scoping reviews, we did not critically appraise the methodological quality or risk of bias of included studies [29]; the largely positive outcomes reported here should therefore be interpreted with appropriate caution, as they have not been weighted or adjusted for study quality.

## Conclusion

A variety of intervention strategies, predominantly delivered through cluster-randomized and individual randomized controlled trials (Table 4), were found to be culturally acceptable, feasible to implement using non-specialist providers, and, in most cases, effective in reducing depressive symptoms or improving psychosocial outcomes for women with PPD in SSA. Results were not uniformly positive; however, a substantial minority of studies, particularly among community health worker-delivered and mother-child dyadic interventions, reported no significant between-group differences in maternal mental health outcomes, underscoring that effectiveness varied by intervention content, delivery model, and population. Very few studies addressed the health system, clinic, community, or provider education level, or incorporated technology-based delivery, and the single intervention identified at the health-system level did not significantly improve its primary outcome; whether interventions at these levels can achieve greater impact than those targeting individual women therefore, remains an open question for future research rather than a finding of this review. We also note that no eligible studies specifically addressed interventions for perinatal anxiety, PTSD, or suicidal ideation. Future scoping and primary research should clarify whether this reflects a genuine gap in intervention research on these conditions in SSA, or a limitation of the currently indexed, English-language, peer-reviewed literature captured by searches such as ours.

## Supporting information

S1 Checklist PRISMA-ScR Checklist. (PDF)

## References

[1] World Health Organization. WHO guide for integration of perinatal mental health in maternal and child health services. 2022. https://www.who.int/publications/i/item/9789240057142

[2] World Health Organization AR. Countries. https://afro.who.int/countries Accessed 2026 February 2.

[3] Gelaye B, Rondon MB, Araya R, Williams MA. Epidemiology of maternal depression, risk factors, and child outcomes in low-income and middle-income countries. Lancet Psychiatry. 2016;3(10):973–82. doi: 10.1016/S2215-0366(16)30284-X 27650773 PMC5155709

[4] Abajobir A, Sidze EM, Wainaina C, Gerbaba MJ, Wekesah FM. The epidemiology of maternal mental health in Africa: a systematic review. Arch Womens Ment Health. 2025;28(5):997–1089. doi: 10.1007/s00737-025-01563-4 40220206 PMC12436556

[5] Agyekum BA. Perceptions and experiences of prenatal mental health: A qualitative study among pregnant women in Ghana. Health Psychol Open. 2023;10(2):20551029231202316. doi: 10.1177/20551029231202316 37720202 PMC10504840

[6] Dadi AF, Akalu TY, Baraki AG, Wolde HF. Epidemiology of postnatal depression and its associated factors in Africa: A systematic review and meta-analysis. PLoS One. 2020;15(4):e0231940. doi: 10.1371/journal.pone.0231940 32343736 PMC7188237

[7] Ajayi AI, Chamdimba E, Sawadogo N, Gitahi N, Tarnagda AM, Ilboudo AK, et al. Socio-ecological factors associated with probable depression among pregnant and parenting adolescent girls: findings from a cross-sectional study in Burkina Faso and Malawi. Reprod Health. 2023;20(1):38. doi: 10.1186/s12978-023-01588-x 36882850 PMC9990966

[8] Cordoba E, Parcesepe AM, Gallis JA, Headley J, Soffo C, Tchatchou B, et al. The syndemic effects of mental ill health, household hunger, and intimate partner violence on adherence to antiretroviral therapy among pregnant women living with HIV in Yaoundé, Cameroon. PLoS One. 2021;16(2):e0246467. doi: 10.1371/journal.pone.0246467 33606692 PMC7894814

[9] Parcesepe AM, Cordoba E, Gallis JA, Headley J, Tchatchou B, Hembling J, et al. Common mental disorders and intimate partner violence against pregnant women living with HIV in Cameroon: a cross-sectional analysis. BMC Pregnancy Childbirth. 2021;21(1):178. doi: 10.1186/s12884-021-03673-0 33663428 PMC7934543

[10] Akorli VV, Adjorlolo S, Puplampu G. Negative life events and maternal mental illness: A study of elite pregnant women in Accra metropolis. Int J Afr Nurs Sci. 2023;19:100634. doi: 10.1016/j.ijans.2023.100634

[11] Atuhaire C, Brennaman L, Cumber SN, Rukundo GZ, Nambozi G. The magnitude of postpartum depression among mothers in Africa: a literature review. Pan Afr Med J. 2020;37:89. doi: 10.11604/pamj.2020.37.89.23572 33244352 PMC7680231

[12] Lasater ME, Murray SM, Keita M, Souko F, Surkan PJ, Warren NE, et al. Integrating Mental Health into Maternal Health Care in Rural Mali: A Qualitative Study. J Midwifery Womens Health. 2021;66(2):233–9. doi: 10.1111/jmwh.13184 33325644 PMC8345229

[13] Hanlon C, Whitley R, Wondimagegn D, Alem A, Prince M. Postnatal mental distress in relation to the sociocultural practices of childbirth: an exploratory qualitative study from Ethiopia. Soc Sci Med. 2009;69(8):1211–9. doi: 10.1016/j.socscimed.2009.07.043 19709793 PMC2791917

[14] Barthel D, Kriston L, Fordjour D, Mohammed Y, Kra-Yao ED, Bony Kotchi CE, et al. Trajectories of maternal ante- and postpartum depressive symptoms and their association with child- and mother-related characteristics in a West African birth cohort study. PLoS One. 2017;12(11):e0187267. doi: 10.1371/journal.pone.0187267 29107975 PMC5673167

[15] Wittkowski A, Gardner PL, Bunton P, Edge D. Culturally determined risk factors for postnatal depression in Sub-Saharan Africa: a mixed method systematic review. J Affect Disord. 2014;163:115–24. doi: 10.1016/j.jad.2013.12.028 24461216

[16] Boisits S, Abrahams Z, Schneider M, Honikman S, Kaminer D, Lund C. Developing a task-sharing psychological intervention to treat mild to moderate symptoms of perinatal depression and anxiety in South Africa: a mixed-method formative study. Int J Ment Health Syst. 2021;15(1):23. doi: 10.1186/s13033-021-00443-5 33722252 PMC7958439

[17] Boisits S. Developing a manualised task-sharing counselling intervention for perinatal common mental disorders in the South African context. University of Cape Town. 2022.

[18] Hoeft TJ, Fortney JC, Patel V, Unützer J. Task-sharing approaches to improve mental health care in rural and other low-resource settings: a systematic review. J Rural Health. 2018;34(1):48–62. doi: 10.1111/jrh.12229 PMC5509535 28084667

[19] Djatche Miafo J, Woks NIE, Nzebou D, Tchaptchet I, Delene ST, Kegha Tchidje O, et al. Epidemiological profile of perinatal mental disorders at a tertiary hospital in Yaoundé- Cameroon. Front Glob Womens Health. 2023;4:999840. doi: 10.3389/fgwh.2023.999840 36817033 PMC9929044

[20] Djatche MJ, Nzebou D, Stoll B, Yimga Ngambia JC, Kouo Ngamby Ekedy M, von Overbeck Ottino S. Validation of the Edinburgh postnatal depression scale and prevalence of depression among adolescent mothers in a Cameroonian context. Sci Rep. 2024;14(1):30670. doi: 10.1038/s41598-024-79370-7 39730398 PMC11681151

[21] Omona K, Babirye D. Maternal near misses (MNM) and their determinants among women who sought obstetric care from fort portal regional referral hospital, Western Uganda. Cogent Public Health. 2022;10(1). doi: 10.1080/27707571.2022.2157996

[22] Fottrell E, Kanhonou L, Goufodji S, Béhague DP, Marshall T, Patel V, et al. Risk of psychological distress following severe obstetric complications in Benin: the role of economics, physical health and spousal abuse. Br J Psychiatry. 2010;196(1):18–25. doi: 10.1192/bjp.bp.108.062489 20044654 PMC2802511

[23] Jarso M, Abdo M, Mannekulih E, Danusa KT, Tefera M, Mezmur H, et al. One in Four Mothers Suffer from Postpartum Depression in Adama Town, Central Oromia Region of Ethiopia: a Cross Sectional Study. Arch Psychiatry Res. 2025;61(1):63–9. doi: 10.20471/jan.2025.61.01.06

[24] Mmasa KN, Liu Y, Jao J, Malee K, Legbedze J, Sun S, et al. Association between maternal HIV status and postpartum depressive symptoms in Botswana. AIDS Care. 2024;:1–6. doi: 10.1080/09540121.2024.2373398 38976581 PMC11707039

[25] Dlamini LP, Hsu Y-Y, Shongwe MC, Wang S-T, Gau M-L. Maternal Self-Efficacy as a Mediator in the Relationship Between Postpartum Depression and Maternal Role Competence: A Cross-Sectional Survey. J Midwifery Womens Health. 2023;68(4):499–506. doi: 10.1111/jmwh.13478 36859814

[26] Ballout S, Darwish SA, Abdelrahman H, Kelly PJ, Aboul-Enein BH. Maternal mental health interventions in the League of Arab states: A scoping review of outcomes and gaps. International Journal of Mental Health. 2025;:1–25. doi: 10.1080/00207411.2025.2566365

[27] Poddar K, Aboul-Enein BH, Lalakia P, Nandagaon V, Khande A, Angadi S, et al. Interventions to address postpartum depression in South Asian countries: a scoping review. J Reprod Infant Psychol. 2026;44(1):204–27. doi: 10.1080/02646838.2025.2464047 39924643

[28] Arksey H, O’Malley L. Scoping studies: towards a methodological framework. Int J Soc Res Methodol. 2005;8(1):19–32. doi: 10.1080/1364557032000119616

[29] Tricco AC, Lillie E, Zarin W, O’Brien KK, Colquhoun H, Levac D, et al. PRISMA Extension for Scoping Reviews (PRISMA-ScR): Checklist and Explanation. Ann Intern Med. 2018;169(7):467–73. doi: 10.7326/M18-0850 30178033

[30] Ouzzani M, Hammady H, Fedorowicz Z, Elmagarmid A. Rayyan-a web and mobile app for systematic reviews. Syst Rev. 2016;5(1):210. doi: 10.1186/s13643-016-0384-4 27919275 PMC5139140

[31] Stansert Katzen L, Tomlinson M, Christodoulou J, Laurenzi C, le Roux I, Baker V, et al. Home visits by community health workers in rural South Africa have a limited, but important impact on maternal and child health in the first two years of life. BMC Health Serv Res. 2020;20(1):594. doi: 10.1186/s12913-020-05436-7 32600455 PMC7325027

[32] Stansert Katzen L, le Roux KW, Almirol E, Hayati Rezvan P, le Roux IM, Mbewu N, et al. Community health worker home visiting in deeply rural South Africa: 12-month outcomes. Glob Public Health. 2021;16(11):1757–70. doi: 10.1080/17441692.2020.1833960 33091320

[33] le Roux IM, Tomlinson M, Harwood JM, O’Connor MJ, Worthman CM, Mbewu N, et al. Outcomes of home visits for pregnant mothers and their infants: a cluster randomized controlled trial. AIDS. 2013;27(9):1461–71. doi: 10.1097/QAD.0b013e3283601b53 23435303 PMC3904359

[34] le Roux KW, Almirol E, Rezvan PH, le Roux IM, Mbewu N, Dippenaar E, et al. Community health workers impact on maternal and child health outcomes in rural South Africa - a non-randomized two-group comparison study. BMC Public Health. 2020;20(1):1404. doi: 10.1186/s12889-020-09468-w 32943043 PMC7496216

[35] Rotheram-Borus MJ, Richter LM, van Heerden A, van Rooyen H, Tomlinson M, Harwood JM, et al. A cluster randomized controlled trial evaluating the efficacy of peer mentors to support South African women living with HIV and their infants. PLoS One. 2014;9(1):e84867. doi: 10.1371/journal.pone.0084867 24465444 PMC3898948

[36] Rotheram-Borus MJ, Tomlinson M, le Roux IM, Harwood JM, Comulada S, O’Connor MJ, et al. A cluster randomised controlled effectiveness trial evaluating perinatal home visiting among South African mothers/infants. PLoS One. 2014;9(10):e105934. doi: 10.1371/journal.pone.0105934 25340337 PMC4207699

[37] Rotheram-Borus MJ, Tomlinson M, Worthman CM, Norwood P, le Roux I, O’Connor MJ. Maternal depression, alcohol use, and transient effects of perinatal paraprofessional home visiting in South Africa: Eight-year follow-up of a cluster randomized controlled trial. Soc Sci Med. 2023;324:115853. doi: 10.1016/j.socscimed.2023.115853 37001280 PMC10121853

[38] Sigwebela S, McConnell BB, Waluwalu N, Davies T, Sanfilippo KRM, Stewart L, et al. Community health intervention through musical engagement (CHIME) in South Africa: A formative exploration of the feasibility and development of a music-based intervention to support perinatal mental health. PLOS Glob Public Health. 2026;6(2):e0004878. doi: 10.1371/journal.pgph.0004878 41662291 PMC12885302

[39] Woollett N, van Eck L, Mazibuko L, Thomson K, Slifko S, Khazin A, et al. Sisonke Ntwana: Describing the mechanisms of change of an arts-based intervention for pregnant and parenting adolescents experiencing violence and poor mental health in South Africa. Child Abuse Negl. 2025;161:107248. doi: 10.1016/j.chiabu.2025.107248 39842249

[40] Cooper PJ, Tomlinson M, Swartz L, Landman M, Molteno C, Stein A, et al. Improving quality of mother-infant relationship and infant attachment in socioeconomically deprived community in South Africa: randomised controlled trial. BMJ. 2009;338:b974. doi: 10.1136/bmj.b974 19366752 PMC2669116

[41] Cooper PJ, Landman M, Tomlinson M, Molteno C, Swartz L, Murray L. Impact of a mother-infant intervention in an indigent peri-urban South African context: pilot study. Br J Psychiatry. 2002;180:76–81. doi: 10.1192/bjp.180.1.76 11772856

[42] Lund C, Schneider M, Garman EC, Davies T, Munodawafa M, Honikman S, et al. Task-sharing of psychological treatment for antenatal depression in Khayelitsha, South Africa: Effects on antenatal and postnatal outcomes in an individual randomised controlled trial. Behav Res Ther. 2020;130:103466. doi: 10.1016/j.brat.2019.103466 31733813 PMC7190434

[43] Seward N, Garman E, Davies T, Hanlon C, MacBeth A, Stewart RC, et al. The importance of social support and food insecurity in improving outcomes for perinatal depression: A causal mediation analysis of the AFFIRM-SA trial. J Affect Disord. 2025;380:552–60. doi: 10.1016/j.jad.2025.03.159 40154807

[44] Spedding M, Stein DJ, Naledi T, Myers B, Cuijpers P, Sorsdahl K. A task-sharing intervention for prepartum common mental disorders: Feasibility, acceptability and responses in a South African sample. Afr J Prim Health Care Fam Med. 2020;12(1):e1–9. doi: 10.4102/phcfm.v12i1.2378 33054272 PMC7564815

[45] Tomlinson M, Rotheram-Borus MJ, le Roux IM, Youssef M, Nelson SH, Scheffler A, et al. Thirty-Six-Month Outcomes of a Generalist Paraprofessional Perinatal Home Visiting Intervention in South Africa on Maternal Health and Child Health and Development. Prev Sci. 2016;17(8):937–48. doi: 10.1007/s11121-016-0676-x 27438294 PMC5111552

[46] Washio Y, Erasmus-Claassen L-A, Taylor SN, Zhang Z, Browne FA, Myers B, et al. An incentive-based text-messaging intervention to reduce maternal alcohol use during pregnancy and lactation in South Africa (MaRISA study): Findings from a single-arm pilot study. Alcohol Clin Exp Res (Hoboken). 2024;48(8):1600–9. doi: 10.1111/acer.15392 38884353

[47] Murnane PM, Bukusi EA, Otieno P, Frongillo EA, Burger RL, Weke E. Effects of an agricultural intervention on psychosocial health among pregnant and non-pregnant women with HIV in Kenya. AIDS. 2025. doi: 10.1097/qad.0000000000004436 41467698 PMC12970629

[48] Eichenauer H, Huss M, Brander M, Bernauer T, Ehlert U. Effects of improved on-farm crop storage on perceived stress and perceived coping in pregnant women-Evidence from a cluster-randomized controlled trial in Kenya. PLoS One. 2023;18(7):e0288446. doi: 10.1371/journal.pone.0288446 37440562 PMC10343033

[49] Green EP, Lai Y, Pearson N, Rajasekharan S, Rauws M, Joerin A, et al. Expanding Access to Perinatal Depression Treatment in Kenya Through Automated Psychological Support: Development and Usability Study. JMIR Form Res. 2020;4(10):e17895. doi: 10.2196/17895 33016883 PMC7573703

[50] Kariuki EW, Kuria MW, Were FN, Ndetei DM. Effectiveness of a brief psychoeducational intervention on postnatal depression in the slums, Nairobi: a longitudinal study. Arch Womens Ment Health. 2021;24(3):503–11. doi: 10.1007/s00737-020-01085-1 33196927

[51] Kim ET, Opiyo T, Acayo PS, Lillie M, Gallis J, Zhou Y, et al. Effect of a lay counselor delivered integrated maternal mental health and early childhood development group-based intervention in Siaya County, Kenya: A quasi-experimental longitudinal study. J Affect Disord. 2021;292:284–94. doi: 10.1016/j.jad.2021.06.002 34134027 PMC8863180

[52] Mutisya RK, Ngure K, Mwachari C. A psychosocial intervention to reduce gender-based violence and antepartum depressive symptoms in pregnant women in Kisumu County, Kenya: a quasi-experimental study. Pan Afr Med J. 2018;29:11. doi: 10.11604/pamj.2018.29.11.13596 29632633 PMC5889516

[53] Notiar A, Jidong DE, Hawa F, Lunat F, Shah S, Bassett P, et al. Treatment of maternal depression in low-income women: A feasibility study from Kilifi, Kenya. Int J Clin Pract. 2021;75(12):e14862. doi: 10.1111/ijcp.14862 34516036

[54] Yator O, John-Stewart G, Khasakhala L, Kumar M. Preliminary Effectiveness of Group Interpersonal Psychotherapy for Young Kenyan Mothers With HIV and Depression: A Pilot Trial. Am J Psychother. 2022;75(2):89–96. doi: 10.1176/appi.psychotherapy.20200050 34915725

[55] Yator O, Khasakhala L, Stewart G-J, Kumar M. Acceptability and impact of group interpersonal therapy (IPT-G) on Kenyan adolescent mothers living with human immunodeficiency virus (HIV): a qualitative analysis. BMC Womens Health. 2022;22(1):240. doi: 10.1186/s12905-022-01807-w 35717156 PMC9206094

[56] Bengtson AM, Filipowicz TR, Mphonda S, Udedi M, Kulisewa K, Meltzer-Brody S, et al. An Intervention to Improve Mental Health and HIV Care Engagement Among Perinatal Women in Malawi: A Pilot Randomized Controlled Trial. AIDS Behav. 2023;27(11):3559–70. doi: 10.1007/s10461-023-04070-8 37084104 PMC10119837

[57] Mhango W, Michelson D, Gaysina D. Feasibility and acceptability of FOotpaths foR adolescent MAternal mental HeAlth (FOR MAMA): A co-designed intervention for pregnant adolescents in Malawi. Glob Ment Health (Camb). 2024;11:e97. doi: 10.1017/gmh.2024.76 39464555 PMC11504933

[58] Ng’oma M, Atif N, Meltzer-Brody S, Chirwa E, Stewart RC. Piloting a psychosocial intervention for perinatal depression, the Thinking Healthy Programme-Peer delivered (THPP), in a primary care setting in Lilongwe District, Malawi. PLOS Glob Public Health. 2024;4(5):e0002128. doi: 10.1371/journal.pgph.0002128 38691572 PMC11062519

[59] Bliznashka L, Nwabuikwu O, Ahun MN, Roschnik N, Phiri B, Gondwe-Matekesa E, et al. Effect of combining lower- and higher-value monthly cash transfers with nutrition-sensitive agriculture, male engagement and psychosocial intervention on maternal depressive symptoms in rural Malawi: a secondary analysis of a cluster-randomised controlled trial. BMJ Glob Health. 2026;11(1):e018860. doi: 10.1136/bmjgh-2025-018860 41500663 PMC12781995

[60] Patil CL, Norr KF, Kapito E, Liu LC, Mei X, Chodzaza E, et al. Group antenatal care positively transforms the care experience: Results of an effectiveness trial in Malawi. PLoS One. 2025;20(6):e0317171. doi: 10.1371/journal.pone.0317171 40531963 PMC12176302

[61] Mhango W, Michelson D, Gaysina D. Co-designing the FOotpaths foR Adolescent MAternal Mental HeAlth (FOR MAMA) intervention for pregnant teens in Malawi. Int Health. 2025;17(4):573–82. doi: 10.1093/inthealth/ihaf007 39912701 PMC12212213

[62] Holmes L, Bitew T, Haile A, Van Lith LM, Burgess S, Vandermark J, et al. Mothers Time: A Cluster Randomized Controlled Trial of the Effects of a Community-Based Cognitive Behavioral Therapy Intervention on Postpartum Mental Health and Family Planning in Northwest Ethiopia. Stud Fam Plann. 2025;56(1):9–39. doi: 10.1111/sifp.70000 40163252

[63] Tessema M, Abera M, Birhanu Z. Antenatal group-based psychoeducation to improve postpartum depression literacy in primary health care institutions in Ethiopia: a cluster randomized controlled trial. Front Psychiatry. 2025;16:1548356. doi: 10.3389/fpsyt.2025.1548356 40313240 PMC12043592

[64] Bitew T, Keynejad R, Sorsdahl K, Myers B, Honikman S, Medhin G, et al. Brief problem-solving therapy (PST) for women experiencing antenatal depressive symptoms: a randomised, controlled feasibility trial in (an) antenatal care setting in rural Ethiopia. Pilot Feasibility Stud. 2025;11(1):121. doi: 10.1186/s40814-025-01672-y 41034949 PMC12486535

[65] Burgess S, Bitew T, Haile A, Souffrant J, Shattuck D, Van Lith LM, et al. The feasibility and acceptability of implementing simplified cognitive behavioral therapy approaches to support postpartum mental health and address associated social and behavioral barriers to postpartum family planning in Amhara, Ethiopia: a qualitative study. BMC Womens Health. 2025;25(1):248. doi: 10.1186/s12905-025-03744-w 40410834 PMC12102818

[66] Lasater ME, Woldeyes GM, Le Roch K, Poku OB, Phan X, Bhardwaj A, et al. Baby Friendly Spaces+: a process evaluation of an integrative health approach for lactating women and their babies in Nguenyyiel refugee camp, Gambella, Ethiopia. Glob Health Action. 2025;18(1):2563422. doi: 10.1080/16549716.2025.2563422 41140190 PMC12570246

[67] Chibanda D, Shetty AK, Tshimanga M, Woelk G, Stranix-Chibanda L, Rusakaniko S. Group problem-solving therapy for postnatal depression among HIV-positive and HIV-negative mothers in Zimbabwe. J Int Assoc Provid AIDS Care. 2014;13(4):335–41. doi: 10.1177/2325957413495564 23881909

[68] Comrie-Thomson L, Webb K, Patel D, Wata P, Kapamurandu Z, Mushavi A, et al. Engaging women and men in the gender-synchronised, community-based Mbereko+Men intervention to improve maternal mental health and perinatal care-seeking in Manicaland, Zimbabwe: A cluster-randomised controlled pragmatic trial. J Glob Health. 2022;12:04042. doi: 10.7189/jogh.12.04042 35596945 PMC9124006

[69] Bliznashka L, Yousafzai AK, Asheri G, Masanja H, Sudfeld CR. Effects of a community health worker delivered intervention on maternal depressive symptoms in rural Tanzania. Health Policy Plan. 2021;36(4):473–83. doi: 10.1093/heapol/czaa170 33313814 PMC8128007

[70] Kaaya S, Siril H, Fawzi MCS, Aloyce Z, Araya R, Kaale A, et al. A peer-facilitated psychological group intervention for perinatal women living with HIV and depression in Tanzania-Healthy Options: A cluster-randomized controlled trial. PLoS Med. 2022;19(12):e1004112. doi: 10.1371/journal.pmed.1004112 36512631 PMC9746973

[71] Saronga HP, Kaaya S, Fawzi MCS. Cost-effectiveness of the Healthy Options group psychosocial intervention for perinatal women living with HIV and depression in Tanzania. PLOS Ment Health. 2024;1(7):e0000066. doi: 10.1371/journal.pmen.0000066 41661746 PMC12798287

[72] Fink G, Melero-Dominguez M, Chembe M, de Vernisy-Romero D, Tembo T, Billima T, et al. Feasibility and acceptability of the Problem Management for Moms programme for improving maternal mental health in Zambia: an open-label trial. Lancet Psychiatry. 2024;11(12):965–74. doi: 10.1016/S2215-0366(24)00256-6 39515361

[73] Spelke MB, Paul R, Blette BS, Meltzer-Brody S, Schiller CE, Ncheka JM, et al. Interpersonal therapy versus antidepressant medication for treatment of postpartum depression and anxiety among women with HIV in Zambia: a randomized feasibility trial. J Int AIDS Soc. 2022;25(7):e25959. doi: 10.1002/jia2.25959 35803896 PMC9270230

[74] Nakku JEM, Nalwadda O, Garman E, Honikman S, Hanlon C, Kigozi F, et al. Group problem solving therapy for perinatal depression in primary health care settings in rural Uganda: an intervention cohort study. BMC Pregnancy Childbirth. 2021;21(1):584. doi: 10.1186/s12884-021-04043-6 34429087 PMC8386083

[75] Wagner GJ, Gwokyalya V, Faherty L, Akena D, Nakigudde J, Ngo V, et al. Effects of M-DEPTH Model of Depression Care on Maternal HIV Viral Suppression and Adherence to the PMTCT Care Continuum Among HIV-Infected Pregnant Women in Uganda: Results from a Cluster Randomized Controlled Trial at Pregnancy Completion. AIDS Behav. 2023;27(9):2902–14. doi: 10.1007/s10461-023-04014-2 36907945 PMC10386969

[76] Poku OB, Becker TD, Rampa S, Misra S, Ho-Foster AR, Entaile P, et al. Theory-Driven, Multi-Stage Process to Develop a Culturally-Informed Anti-Stigma Intervention for Pregnant Women Living with HIV in Botswana. Int J MCH AIDS. 2022;11(2):e569. doi: 10.21106/ijma.569 36320928 PMC9617017

[77] Yang LH, Eschliman EL, Mehta H, Misra S, Poku OB, Entaile P, et al. A pilot pragmatic trial of a “what matters most”-based intervention targeting intersectional stigma related to being pregnant and living with HIV in Botswana. AIDS Res Ther. 2022;19(1):26. doi: 10.1186/s12981-022-00454-3 35739534 PMC9219368

[78] Agler RA, Zivich PN, Kawende B, Behets F, Yotebieng M. Postpartum depressive symptoms following implementation of the 10 steps to successful breastfeeding program in Kinshasa, Democratic Republic of Congo: A cohort study. PLoS Med. 2021;18(1):e1003465. doi: 10.1371/journal.pmed.1003465 33428617 PMC7799755

[79] Bah AJ, Wurie HR, Samai M, Horn R, Ager A. Feasibility, acceptability and preliminary effectiveness of a culturally adapted nonspecialist delivery Problem-Solving Therapy: Friendship Bench Intervention for perinatal psychological distress in Sierra Leone. Glob Ment Health (Camb). 2025;12:e16. doi: 10.1017/gmh.2025.6 40028388 PMC11867829

[80] Balogun OO, Aoki A, Tomo CK, Mochida K, Fukushima S, Mikami M, et al. Effectiveness of the maternal and child health handbook for improving continuum of care and other maternal and child health indicators: A cluster randomized controlled trial in Angola. J Glob Health. 2023;13:04022. doi: 10.7189/jogh.13.04022 36730071 PMC9894505

[81] Baumgartner JN, Ali M, Gallis JA, Lillie M, Owusu R, Abubakr-Bibilazu S, et al. Effect of a lay counselor-delivered integrated maternal mental health and early childhood development group-based intervention in Northern Ghana: a cluster-randomized controlled trial. Glob Ment Health (Camb). 2021;8:e18. doi: 10.1017/gmh.2021.15 34104458 PMC8157813

[82] Callands TA, Hylick K, Desrosiers A, Gilliam SM, Taylor EN, Hunter JJ, et al. The feasibility and acceptability of Project POWER: a mindfulness-infused, cognitive-behavioral group intervention to address mental and sexual health needs of young pregnant women in Liberia. BMC Pregnancy Childbirth. 2023;23(1):196. doi: 10.1186/s12884-023-05435-6 36941545 PMC10026523

[83] Dozio E, Le Roch K, Bizouerne C. Baby friendly spaces: an intervention for pregnant and lactating women and their infants in Cameroon. Intervention. 2020;18(1):78. doi: 10.4103/intv.intv_61_18

[84] Gureje O, Oladeji BD, Kola L, Bello T, Ayinde O, Faregh N, et al. Effect of intervention delivered by frontline maternal care providers to improve outcome and parenting skills among adolescents with perinatal depression in Nigeria (the RAPiD study): A cluster randomized controlled trial. J Affect Disord. 2022;312:169–76. doi: 10.1016/j.jad.2022.06.032 35752215

[85] McKinnon B, Sall M, Vandermorris A, Traoré M, Lamesse-Diedhiou F, McLaughlin K, et al. Feasibility and preliminary effectiveness of group antenatal care in Senegalese health posts: a pilot implementation trial. Health Policy Plan. 2020;35(5):587–99. doi: 10.1093/heapol/czz178 32155254

[86] Sanfilippo KRM, McConnell B, Cornelius V, Darboe B, Huma HB, Gaye M, et al. Community psychosocial music intervention (CHIME) to reduce antenatal common mental disorder symptoms in The Gambia: a feasibility trial. BMJ Open. 2020;10(11):e040287. doi: 10.1136/bmjopen-2020-040287 33234641 PMC7684808

[87] Bam VB, Diji AK-A, Lartey SD, Nabirye R, Owusu-Ansah FE, Lomotey AY, et al. Feasibility and effectiveness of an interprofessional educational program on perinatal mental health: perspectives of health students in two African countries. BMC Med Educ. 2026;26(1):199. doi: 10.1186/s12909-025-08554-0 41501756 PMC12869977

[88] Pan J, Luo W, Zhang H, Wang Y, Lu H, Wang C, et al. The Effects of Online Cognitive Behavioral Therapy on Postpartum Depression: A Systematic Review and Meta-Analysis. Healthcare (Basel). 2025;13(7):696. doi: 10.3390/healthcare13070696 40217993 PMC11989130

[89] Barker PM, Reid A, Schall MW. A framework for scaling up health interventions: lessons from large-scale improvement initiatives in Africa. Implement Sci. 2016;11:12. doi: 10.1186/s13012-016-0374-x 26821910 PMC4731989

[90] Bulthuis SE, Kok MC, Raven J, Dieleman MA. Factors influencing the scale-up of public health interventions in low- and middle-income countries: a qualitative systematic literature review. Health Policy Plan. 2020;35(2):219–34. doi: 10.1093/heapol/czz140 31722382 PMC7050685

[91] Rahman A, Malik A, Sikander S, Roberts C, Creed F. Cognitive behaviour therapy-based intervention by community health workers for mothers with depression and their infants in rural Pakistan: a cluster-randomised controlled trial. Lancet. 2008;372(9642):902–9. doi: 10.1016/S0140-6736(08)61400-2 18790313 PMC2603063

[92] Chowdhary N, Sikander S, Atif N, Singh N, Ahmad I, Fuhr DC, et al. The content and delivery of psychological interventions for perinatal depression by non-specialist health workers in low and middle income countries: a systematic review. Best Pract Res Clin Obstet Gynaecol. 2014;28(1):113–33. doi: 10.1016/j.bpobgyn.2013.08.013 24054170 PMC3893480

[93] Dennis C-L, Dowswell T. Psychosocial and psychological interventions for preventing postpartum depression. Cochrane Database Syst Rev. 2013;2013(2):CD001134. doi: 10.1002/14651858.CD001134.pub3 23450532 PMC11936315

[94] Kumar D, Hameed W, Avan BI. Comparing the effectiveness of mother-focused interventions to that of mother-child focused interventions in improving maternal postpartum depression outcomes: A systematic review. PLoS One. 2023;18(12):e0295955. doi: 10.1371/journal.pone.0295955 38117801 PMC10732407

[95] Özcan NK, Boyacıoğlu NE, Dinç H. Postpartum Depression Prevalence and Risk Factors in Turkey: A Systematic Review and Meta-Analysis. Arch Psychiatr Nurs. 2017;31(4):420–8. doi: 10.1016/j.apnu.2017.04.006 28693880

[96] Edwards LM, Le H-N, Garnier-Villarreal M. A Systematic Review and Meta-Analysis of Risk Factors for Postpartum Depression Among Latinas. Matern Child Health J. 2021;25(4):554–64. doi: 10.1007/s10995-020-03104-0 33394276

[97] Alshikh Ahmad H, Alkhatib A, Luo J. Prevalence and risk factors of postpartum depression in the Middle East: a systematic review and meta-analysis. BMC Pregnancy Childbirth. 2021;21(1):542. doi: 10.1186/s12884-021-04016-9 34362325 PMC8343347

[98] Dosani A, Arora H, Mazmudar S. mHealth and perinatal depression in low-and middle-income countries: a scoping review of the literature. Int J Environ Res Public Health. 2020;17(20). doi: 10.3390/ijerph17207679 33096738 PMC7589927

[99] Sun X, Wang R, Cong S, Fan X, Sha L, Feng J, et al. Effect of music intervention on perinatal depressive symptoms: A meta-analysis. J Psychiatr Res. 2024;178:78–87. doi: 10.1016/j.jpsychires.2024.08.004 39126879

[100] Brown JVE, Wilson CA, Ayre K, Robertson L, South E, Molyneaux E, et al. Antidepressant treatment for postnatal depression. Cochrane Database Syst Rev. 2021;2(2):CD013560. doi: 10.1002/14651858.CD013560.pub2 33580709 PMC8094614

[101] Edhi M, Abid M, Khemane Z, Asif M, Mustafa MS, Rangwala HS, et al. Postpartum Depression: Role of Therapy and Associated Stigmas in Developing Countries. Ann Neurosci. 2024;31(1):10–1. doi: 10.1177/09727531231203453 38584985 PMC10996872

[102] Elshatarat RA, Yacoub MI, Saleh ZT, Ebeid IA, Abu Raddaha AH, Al-Za’areer MS, et al. Perinatal Nurses’ and Midwives’ Knowledge About Assessment and Management of Postpartum Depression. J Psychosoc Nurs Ment Health Serv. 2018;56(12):36–46. doi: 10.3928/02793695-20180612-02 29916523

[103] Moore Simas TA, Flynn MP, Kroll-Desrosiers AR, Carvalho SM, Levin LL, Biebel K, et al. A Systematic Review of Integrated Care Interventions Addressing Perinatal Depression Care in Ambulatory Obstetric Care Settings. Clin Obstet Gynecol. 2018;61(3):573–90. doi: 10.1097/GRF.0000000000000360 29553986 PMC6059986

[104] Wang T-H, Tzeng Y-L, Teng Y-K, Pai L-W, Yeh T-P. Evaluation of psychological training for nurses and midwives to optimise care for women with perinatal depression: a systematic review and meta-analysis. Midwifery. 2022;104:103160. doi: 10.1016/j.midw.2021.103160 34753017

